# A compliance assessment tool for household pit latrines and cesspits against RS ISO 24521: evidence from Kigali City

**DOI:** 10.3389/fpubh.2026.1795680

**Published:** 2026-05-29

**Authors:** Marie Leonce Murebwayire, Erik Nilsson, Umaru Garba Wali, Innocent Nhapi

**Affiliations:** 1 Department of Civil, Environmental, and Geomatic Engineering, School of Engineering, College of Science and Technology, University of Rwanda, Kigali, Rwanda; 2 Department of Building and Environmental Technology, Division of Water Resources Engineering, Faculty of Engineering, Lund University, Lund, Sweden; 3 United Nations University Hub on Water in a Changing Environment (WICE) at Lund University, United Nations University Institute for Water, Environment and Health (UNU-INWEH), Lund, Sweden; 4 Centre for Urban Resilience, Water and Climate Change, Chinhoyi, Zimbabwe

**Keywords:** assessment tool, cesspit, compliance, income, ISO 24521, pit latrines

## Abstract

**Introduction:**

On-site sanitation systems (OSS) are the primary sanitation technology used to manage household fecal sludge in the Global South, including Kigali City. Poor quality OSS can lead to the spread of fecal-oral diseases and water resource pollution. Previous studies have demonstrated that OSS in Kigali are ineffective. In 2018, the Rwanda Standards Board adopted ISO 24521 to guide the design, construction, and management of domestic OSS. However, limited evidence exists on the compliance of OSS with the established guidelines, and no established tools are currently available to assess such compliance. This study developed and tested an assessment tool to evaluate the compliance of households’ pit latrines and cesspits against RS ISO 24521 and identify factors associated with compliance. The study further proposes recommendations for enhanced compliance coverage and safe sanitation services in Kigali.

**Methods:**

The tool was tested through a survey of 903 households in five sectors of Gasabo District in Kigali, through facility inspection, questions, and observations. Data were analyzed using descriptive statistics, Generalized Linear Mixed Model, and Rao-Scott chi-squares. Within RS ISO 24521, four standard objectives were covered: (i) Protection of Public Health, (ii) Meeting Needs and Expectations of Users, (iii) Sustainability, and (iv) Protection of Environment.

**Results:**

The results show that full compliance with the four standard objectives was 0.5, 4.8, 0.4 and 3.4% for pit latrines, and 79.2, 11, 0.4 and 2% for cesspits. Technical awareness and hygiene behavioral factors were found to be associated with low compliance. Both pit latrines and cesspits showed very low full compliance with the Sustainability objective, which in turn precluded examination of any associations to full compliance for this objective. For characteristics included in Protection of Public Health, factors such as wealth, house tenure, and residence type showed significant associations.

**Discussion and conclusion:**

To address the low compliance with selected objectives of RS ISO24521, recommendations emphasize increased enforcement of regulations, sanitary inspections, promotion of hygiene behavior change, and sustainable water supply. By evaluating the assessment tool, an improved version is proposed to support the management of OSS in Kigali, as well as in other cities with rapid urban growth and high reliance on OSS.

## 1 Introduction

As per the Sustainable Development Goal number 6, by 2030, everyone and everywhere in the World is expected to have access to and use an improved toilet where waste will be safely managed. In 2010, access to adequate sanitation was declared a human right and fundamental for public health, setting the stage for governments to accelerate efforts to achieve 100% coverage of safely managed sanitation (86) (1). Globally, progress toward safely managed sanitation is slow. As of 2024, about 42% of the global population, against 74% in sub-Saharan Africa, lacked access to safely managed sanitation (2). In many low- and middle-income countries, onsite sanitation facilities remain the primary sanitation facilities in use due to limited sewered systems. On-site sanitation systems (OSSs) are considered safely managed when they are improved, not shared, containments store and treat fecal sludge *in situ*, or ensure the safe separation of human excreta from human contact along the service chain from toilet interface/collection and containment to final disposal or end use (2, 3). According to a recent JMP report, in some middle and low-income countries where the prevalence of onsite sanitation is high, some were found to be safely managed, while others had poor containment or were subjected to events such as collapse, flooding, or overflowing (2). In developing countries, urban settings are growing at a higher pace than the sanitation services, leading to more people with limited or no access to sanitation (4). Globally, from 2020 to 2024, people sharing toilets increased by a third, mainly in urban settings (2).

Lack of proper sanitation in urban informal settlements, coupled with severe climate events such as floods, can increase risks of exposure to pathogens (4) and contaminate water resources and soil (5). Vulnerable populations, such as children, are more affected, with negative health outcomes such as stunting and death (3). Globally, mortality rates due to sanitation and hygiene-related diseases among children under 5 years old resulted in 395,000 deaths in 2019 (6).

In Kigali, the capital city of Rwanda, approximately 80% of residents rely on pit latrines, about 15% use flush toilets, of which only about 7% are served by decentralized wastewater systems (7, 8). Data on safely managed sanitation services coverage in Kigali remain scarce, near nonexistent (2, 8). Among those using flush toilets, the fecal sludge is discharged either into septic tanks or into cesspits. Despite the high use of pit latrines and cesspits, a limited number of households use professional emptying services; others opt to seal full pits and dig new ones, perform emptying by themselves, or hire unprofessional sanitation workers (9), increasing risks of contamination. Additionally, part of 60% of the population living in unplanned settlements is located in areas inaccessible to mechanized emptying trucks, requiring the use of intensive labor methods, which increase the costs of the service (9–11). Recent studies have reported operational challenges associated with pit latrines in Kigali, including poor quality of construction, unhygienic conditions, limited access for emptying services, and the presence of solid waste in pits (10, 12–14). Moreover, the construction and operation of pit latrines often do not follow established guidelines, posing risks of collapse, spread of poor sanitation-related diseases, environmental pollution, and reduced privacy (12, 15, 16).

The extensive reliance on these types of OSSs with quality issues may be related to the notable prevalence of sanitation and hygiene-related diseases. According to a recent health demographic survey in Kigali, the prevalence of diarrheal disease among consulted children was about 14% (17) and stunting 27% (18), while the overall Soil Transmitted Helminthiasis (STH) prevalence is about 38% in Rwanda (85).

In 2019, the government of Rwanda, through the Rwanda Standard Board (RSB), adopted the ISO 24521 as a national standard (RS ISO 24521) to provide guidelines ensuring that the design, construction, and management of basic onsite domestic wastewater services are safe, hygienic, and environmentally friendly (19). However, there is limited knowledge on the extent to which households’ OSSs comply with this standard in Kigali and the factors that may be linked to compliance. Furthermore, there is a lack of established tools to assess compliance. In addition, most previous studies have focused on informal settlements and pit latrines, leaving cesspits and other settlements unassessed. Developing assessment tools and assessing the compliance levels are thus urgently needed, not only to generate evidence on progress toward SDG 6.2, but also to enable development and application of context-adapted strategies, on-site sanitation management, and monitoring to increase compliance and promote environmental health.

In light of these OSS conditions and related knowledge gaps, this study aims to develop and test a tool to assess compliance with the RS ISO 24521 of households’ pit latrines and cesspits in Kigali. In addition, the study aims to analyze factors associated with full compliance with this standard. The findings are expected to provide critical insights to enhance household sanitation service delivery and refine the proposed assessment tool for greater applicability and scalability. By assessing the compliance levels of pit latrines and cesspits with RS ISO24521, this study contributes to the establishment of a baseline dataset on the coverage of safely managed sanitation services in Kigali. In addition, the study initiates the first RS ISO 24521 compliance assessment tool for pit latrines and cesspits in Rwanda. The findings of this study are thus particularly relevant for sanitation governance in Kigali, Rwanda, and more broadly for rapidly urbanizing contexts characterized by extensive reliance on domestic OSS similar to those in Kigali.

## 2 Materials and methods

### 2.1 Study design

**Figure 1:**
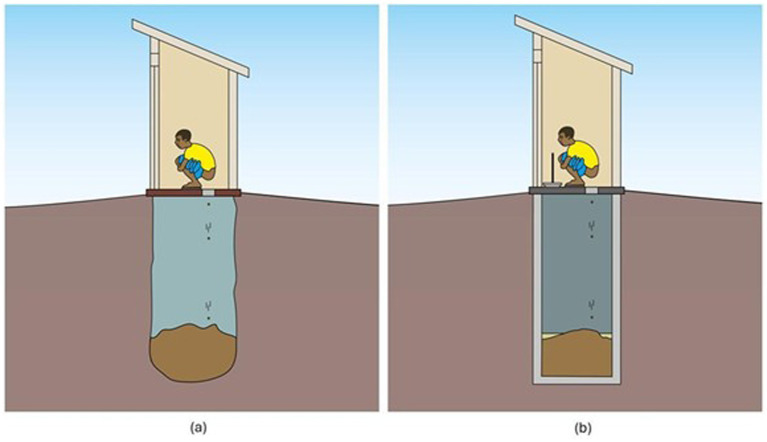
**(a)** Unlined pit latrine (76, CC BY-NC 4.0, Loughborough University Research Repository); **(b)** Completely lined pit latrine (77, CC BY-NC 4.0, Loughborough University Research Repository).

**Figure 2:**
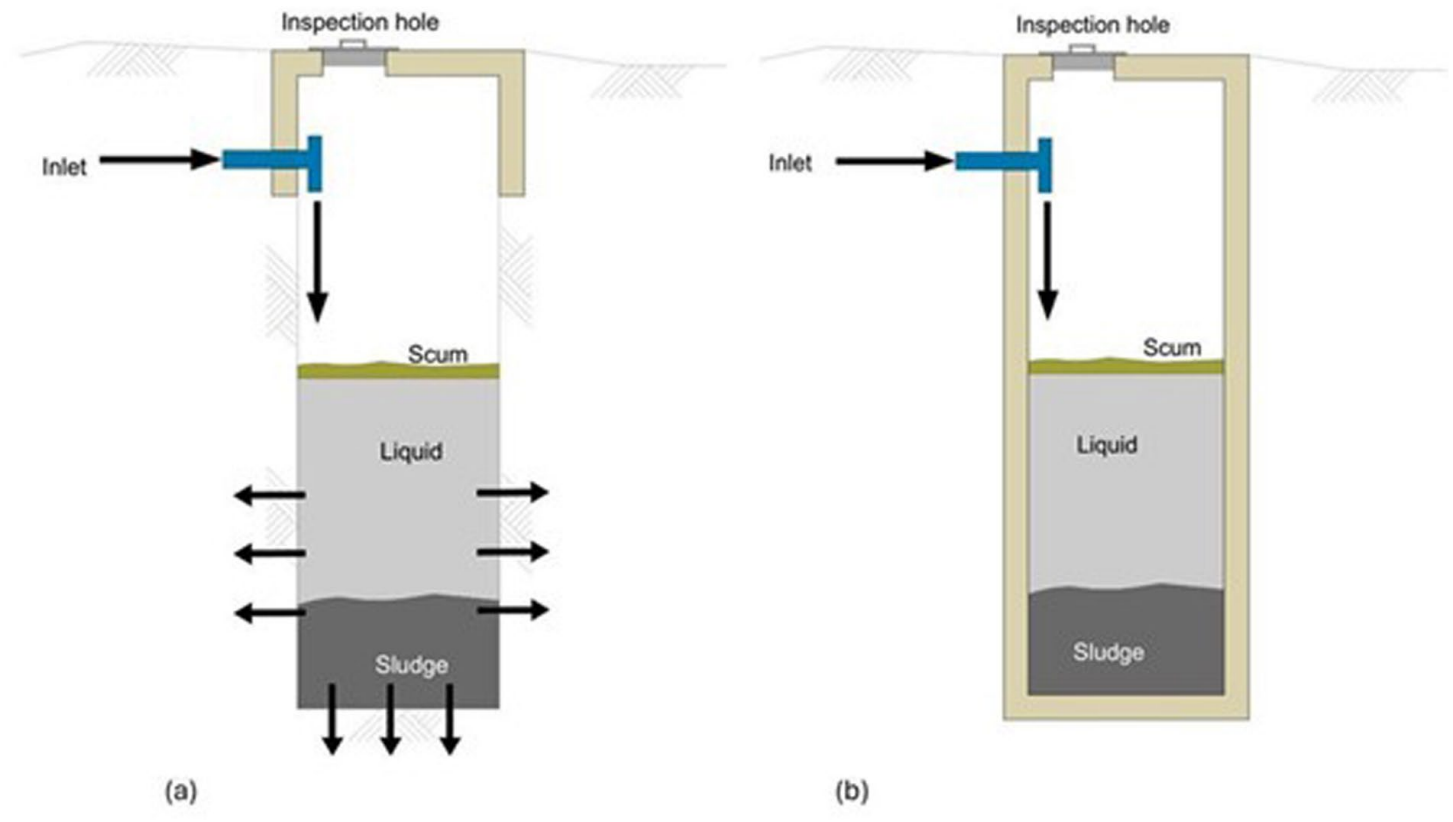
**(a)** Unlined cesspit, **(b)** Lined cesspits. Adapted from (20). CC-BY-SA 3.0 IGO. Global Water Pathogens Project.

This study focuses on assessing the compliance of pit latrines and cesspits, which are the onsite sanitation facilities used by about 80% of households in Kigali. Pit latrines are referred to as lined or unlined pits with a superstructure for user interface, where a lined pit has walls and a bottom sealed (Figure 1). Cesspits are lined or unlined pits with a cover, into which wastewater from flushing toilets is discharged, and are generally located far from the user interface (Figure 2) (20, 21). As the operational definition adopted for the purpose of this study, a lined pit is defined as having fully sealed walls and bottom, in accordance with RURA (2020) and the Compendium of Sanitation Systems and Technologies (21).

In Rwanda, a household is defined as a group of people sharing one housing, living and eating together, related or not by blood or marriage (22). In this study, a compound is defined to designate a plot, fenced or not, comprising one or more households. New settlements are defined as settlements constructed following the Kigali master plan, while old settlements refer to settlements constructed before or not in accordance with the implementation of the master plan. Income levels were categorized based on the socioeconomic mapping program named *Ubudehe*, used by the government of Rwanda to leverage social capital in social protection enhancement (23, 24, 81).

#### 2.1.1 Development and testing of the tool

To generate detailed data on the condition of pit latrines and cesspits, a cross-sectional study was conducted using a specifically developed and tested tool that combined household surveys, sanitation facility inspections, and direct observation. The tool intended to assess the compliance of pit latrines and cesspits with ISO 24521, while helping to identify critical gaps in compliance and informing recommendations for enhanced household sanitation services The survey was designed to align with RS ISO 2452, by setting and adapting standard compliance indicators based on the Rwanda Utilities and Regulatory Authority’s Guidelines for Fecal Sludge Management (RURA, 2020) and the WHO Sanitary Inspection questionnaire (25).

The tool assesses four selected objectives of RS 24521, namely: (1) Protection of Public Health, (2) Meeting Needs and Expectations of Users, (3) Sustainability of Basic Onsite Domestic Wastewater Systems (referred to as Sustainability from hereon), and (4) Protection of Environment. For each objective, a set of compliance indicators, herein referred to as characteristics, were proposed, as detailed in Table 1. These characteristics were derived by systematically mapping each requirement of the four selected RS ISO 24521 objectives to measurable indicators, drawing additionally on the WHO Sanitary Inspection questionnaire (25) and the RURA Guidelines for Fecal Sludge Management (87). Table 1 outlines the type of information gathered, categorized into characteristics to assess features of the standard, related demographic information, and recommendations by respondents. Table 1 outlines the survey response options and the characteristics assigned to pit latrines and cesspits. The decision to assign specific characteristics to pit latrines or cesspits was based on structural differences between the two facility types and local context, as further explained in section 2.1.2. Table 2 presents details on the number of characteristics assigned to each selected specific objective and facility type. The same table also describes the compliance scoring framework, which is further explained in section 2.4. All this information was collected to provide a comprehensive understanding of the facilities’ compliance and the relationship between characteristics. A total set of 26 characteristics was identified as potential indicators for the four selected objectives (Tables 1, 2).

**Table 1:** Investigated characteristics in the survey.

Topic	Characteristic *Question **Observation	Operational definition	Measurement scale	Standard compliance criteria	Source	Measured for Pit latrines (Yes/No)	Measured for Cesspits (Yes/No)
Standard objectives							
(1) Protection of Public Health	Evidence of leaking into the surrounding environment**	No visible leakages from the pit or slab to external environment	Yes, No	No occurrence	Adapted from WHO^1^, RURA^2^ and RS ISO 24521^3^	Yes	No
	Cleanliness of the interface (only for pit latrine) and the pit surface**	No visible fecal matter and urine, no stagnant water, and no solid waste around the pit and in the interface	Clean, Fairly clean to very dirty	Clean	Adapted from WHO, RURA, and RS ISO 24521	Yes	Yes
	Presence of flies**	Presence of insect in or around the facility	No flies, Few flies, Many flies	No flies	Adapted from WHO, RURA, and RS ISO 24521	Yes	Yes
	Pit smell**	Strong fecal smell	No bad smell, Slight to strong bad smell	No bad smell	Adapted from WHO, RURA, and RS ISO 24521	Yes	Yes
	Presence of handwashing (for Pit Latrine)**	Handwashing close to the pit latrine	Yes, No	Yes	Adapted from WHO and RURA	Yes	No
	Presence of piped water in the compound **	Water tap in the compound	Yes, No	Yes	Authors’ suggestions and adapted from WHO	No	Yes
	Presence of gas stinking**	Gas stinking in eyes or gas overlapping air	No gas smell, Moderate to strong gas smell	No gas smell	Authors’ suggestions and adapted from RS ISO 24521	Yes	Yes
(2) Meeting Needs and Expectations of Users	Facility provides security to intended users**	No visible collapse risk, no overly exposed pit opening, functional door, privacy ensured	Yes, No	Yes	Adapted from WHO and RS ISO 24521	Yes	Yes
	Facility sharing status*	The facility is shared with other households	Shared, Not shared	Not shared	Adapted from WHO and RS ISO 24521	Yes	Yes
	Users’ satisfaction with facility*	Users are satisfied with the facility	Yes, No	Yes	Adapted from RS ISO 24521	Yes	Yes
	Danger of pit to other houses **	Leaking toward or risks of collapsing over other houses	Yes, No	No	Adapted from WHO and RS ISO 24521	Yes	Yes
	Reasons for choosing the type of sanitation facility*	Motivations to choose the type of facility	Affordable, Commonly used, Advised by a technician	Affordable	Adapted from RS ISO 24521	Yes	Yes
(3) Sustainability of basic onsite domestic wastewater systems	Type of slab**	Pit cover	Reinforced concrete, Timber, Other	Reinforced concrete	Adapted from RURA and RS ISO 24521	Yes	Yes
	Pit lining status*	Pit’s walls and bottom are sealed	Lined, Partially Lined, Unlined	Lined	Adapted from WHO, RURA and RS ISO 24521	Yes	Yes
	Conditions of superstructure **	No visible cracks or structural damage on superstructure	Good condition, bad to very bad condition	Good Condition	Adapted from WHO, RURA and RS ISO 24521	Yes	No
	Conditions of the slab and pit**	No visible cracks or structural damage on slab	Good condition, bad to very bad condition	Good Condition	Authors’ suggestion and adapted from WHO, RURA	Yes	Yes
	Frequency of emptying*	How many times the pit was emptied since its construction	New, At least once, Never	New or At least once	Adapted from RURA and RS ISO 24521	Yes	Yes
	Rainwater drainage into the pit**	Visible rainwater path into the pit	Yes, No	No	Adapted from RURA and RS ISO 24521	Yes	Yes
	Type of effluents and waste discharge into pit*	Other type of wastewater or waste disposed of into the facility	Only excreta or blackwater, Domestic trash, Laundry, Kitchen Water	Only excreta or blackwater	Adapted from RURA and RS ISO 24521	Yes	Yes
	Presence of an opening for emptying services**	covered manhole for emptying services	Yes, No	Yes	Adapted from RURA and RS ISO 24521	Yes	Yes
	Accessibility of the residence by a road at least 3 m wide**	The household’s accessibility by a road of 3 m minimum	Yes, No	Yes	Authors’ suggestion and adapted from RURA and RS ISO 24521	Yes	Yes
	Accessibility of the pit for emptying services**	The pit can be accessed easily by exhauster’s pipe	Yes, No	Yes	Adapted from WHO, RURA and RS ISO 24521	Yes	Yes
	Knowledge of regulations about toilet construction in Kigali City*	Interviewed person knows regulations on toilet construction	Yes, No	Yes	Authors’ suggestion and adapted from RURA and RS ISO 24521	Yes	Yes
(4) Protection of environment	Depth of the pit*	How deep is the pit	Less than 10 m, Between 10 and 20 m, Over 20 m	Less than 10 m	Authors’ suggestion and adapted from RURA and RS ISO 24521	Yes	Yes
	Access to groundwater or moist soil when digging the pit*	Groundwater or moist soil was reached when the pit was being dug	Groundwater reached, Moist soil reached, No water reached	No water reached	Authors’ suggestion and adapted from RURA and RS ISO 24521	Yes	Yes
	Sanitary inspections by authorities*	Authorities came to inspect the facility at least once	Yes, No	Yes	Adapted from WHO and RS ISO 24521	Yes	Yes
Demographic and other information							
Demographic	Wealth of household*	As per social category framework in Rwanda^4^	Very Low, Low, Middle, High	NA	Authors’ suggestion	Yes	Yes
	Residential type*	Living in a compound shared by many households or in a single family house	Compound with many households, Single house	NA	Authors’ suggestion	Yes	Yes
	Type of settlement**	Settlements of before and as per city masterplan	Old, New	NA	Authors’ suggestion	Yes	Yes
	House tenure*	Household owns the house where they live or they are tenants	Owning, Renting	NA	Authors’ suggestion	Yes	Yes
	Presence of piped water in the compound*/**	Piped water tap in the compound	Yes, Non	NA	Authors’ suggestions and adapted from WHO	Yes	Yes
	Age of pit*	When the pit/facility was constructed	Less than 5 years, between 5 and 10 years ago, More than 10 years	NA	Authors’ suggestion	Yes	Yes
Other	Number of households sharing one facility*	Number of households sharing one pit*	Number	NA	Adapted from WHO	Yes	Yes
	Presence of other pits in the compound for stormwater and other households’ wastewater*	whether there are other pits used to contain other type of household wastewater	Yes, No	NA	Authors’ suggestion	Yes	Yes
	Recommendations for improved sanitation services*	Suggestions from the household on what the government could do to improve sanitation	Open question	NA	Authors’ suggestion	Yes	Yes

^1^RURA (2020); ^2^WHO (25); ^3^RSB (19); ^4^Karekezi and Turatsinze (24).

**Table 2:** Scoring framework for compliance of pit latrines and cesspits with selected objectives of RS ISO 24521.

Selected standard objectives	Total number of investigated characteristics	Pit latrines			Cesspits		
		Number of investigated characteristics	Number of characteristics complying	Compliance level	Number of investigated characteristics	Number of characteristics complying	Compliance level
(1) Protection of Public Health	7	6	6	Full Compliance	5	5	Full Compliance
			4–5	Moderate Compliance		3–4	Moderate Compliance
			1–3	Low Compliance		1–2	Low Compliance
			0	Non-Compliance		0	Non-Compliance
(2) Meeting Needs and Expectations of Users	5	5	5	Full Compliance	5	5	Full Compliance
			3–4	Moderate Compliance		3–4	Moderate Compliance
			1–2	Low Compliance		1–2	Low Compliance
			0	Non-Compliance		0	Non-Compliance
(3) Sustainability of basic onsite domestic wastewater systems	11	11	11	Full Compliance	10	10	Full Compliance
			6–10	Moderate Compliance		5–9	Moderate Compliance
			1–5	Low Compliance		1–4	Low Compliance
			0	Non-Compliance		0	Non-Compliance
(4) Protection of the environment	3	3	3	Full Compliance	3	3	Full Compliance
			2	Moderate Compliance		2	Moderate Compliance
			1	Low Compliance		1	Low Compliance
			0	Non-Compliance		0	Non-Compliance

For each of the four selected objectives of the RS ISO 24521, characteristics were developed and assigned to assess the main features of the selected standard objective, based on the requirements for basic OSS that describe each objective as per RS ISO 24. These characteristics were operationalized based on the WHO Sanitary Inspection questionnaire (25) and the RURA Guidelines for Fecal Sludge Management (RURA, 2020). The selected objectives are described as follows:

1. *Protection of Public Health*: preventing direct human exposure to excreta, inaccessibility of excreta by vectors and insects, protection from odor or aesthetic nuisance, and protection of drinking water supplies. Seven (7) characteristics were developed under this objective, of which six (6) were used for pit latrines and five (5) for cesspits (Tables 1, 2).
2. *Meeting Needs and Expectations of Users*: covering comfort, satisfaction, affordability, and safety. Five (5) characteristics were developed under this objective and assigned to both pit latrines and cesspits (Tables 1, 2).
3. *Sustainability*: the capability to meet current and future needs, planned regular desludging, properly maintained, and designed with safety considerations. Eleven characteristics were developed under this objective, all assigned to pit latrines and only ten assigned to cesspits (Tables 1, 2).
4. *Protection of Environment*: onsite evaluation by competent authorities, preventing accumulation of wastewater on the ground and percolation into groundwater. Three (3) characteristics were developed under this objective, and all were assigned to both pit latrines and cesspits (Tables 1, 2).

The difference in the number of characteristics assigned to pit latrines and cesspits results in a partial comparison between the two types of facilities. The RS ISO 24521 also includes the following objectives, which were not covered in this study as they are not directly related to requirements for basic onsite systems: Protection of Users and Operators, Provision of Services under Normal Conditions and Emergencies, and Promotion of Sustainable Development of the Community.

Other information related to how households manage stormwater and other onsite-generated wastewater was also collected to deepen the understanding of household onsite wastewater management. Information in the survey was collected either by (i) observations by the enumerator, independently of respondent statements, minimizing recall and social desirability bias, or (ii) self-reported via structured interview and therefore subject to greater susceptibility to response bias. Observations by enumerators were carried out through physical inspection of the sanitation facility.

#### 2.1.2 Internal refinement and pilot testing of the tool

The tool is derived from a combination of aspects from RS ISO 24521, WHO Sanitary Inspection, and Fecal Sludge Management Guidelines (RURA, 2020). During the tool development phase, consultations were held with subject matter experts in Water, Sanitation and Hygiene (WASH), Environmental Engineering, Water Resources, Environmental Health, and Public Health, as well as Sanitation Entrepreneurs. Their insights were integrated into the tool, particularly regarding the assessment of structural components of sanitation facilities, operation practices, public health considerations, and pathways of environmental pollution.

The team of enumerators consisted of six students in their final year of BSc in Environmental Health at University of Rwanda (UR), who also contributed to the practical adaptation of the tool. Through interactive sessions, including site visits to representative facilities and visualization of various similar facilities, enumerators were trained on the survey, how to inspect the facility, and observe targeted characteristics. A pilot testing session was conducted with about 35 responses, which were not included in the final responses. This led the research team and enumerators to decide on approaches for interpreting observed data. For example, some characteristics were investigated for pit latrines but not for cesspits, and vice versa. For instance, handwashing stations and facility superstructure were only investigated for pit latrines, as these are components mandatory only for pit latrines. The leaking condition of cesspits was not investigated as both the research and enumerators teams agreed that, given the local context and the nature of cesspits, such observations carried a high risk of misinterpretation. The presence of piped water was investigated only for cesspits because access to water is a basic prerequisite for flushing toilets. In contrast to cesspits, the presence of piped water was not investigated for pit latrines because it is not an operational requirement for this type of sanitation facility. Similarly, the condition of the superstructure was not investigated for cesspits because these are generally located away from the user interface and do not have a superstructure. In addition, operational definitions detailed in Table 1 were agreed upon to ensure reproducibility across enumerators and sites.

### 2.2 Study area

**Figure 3:**
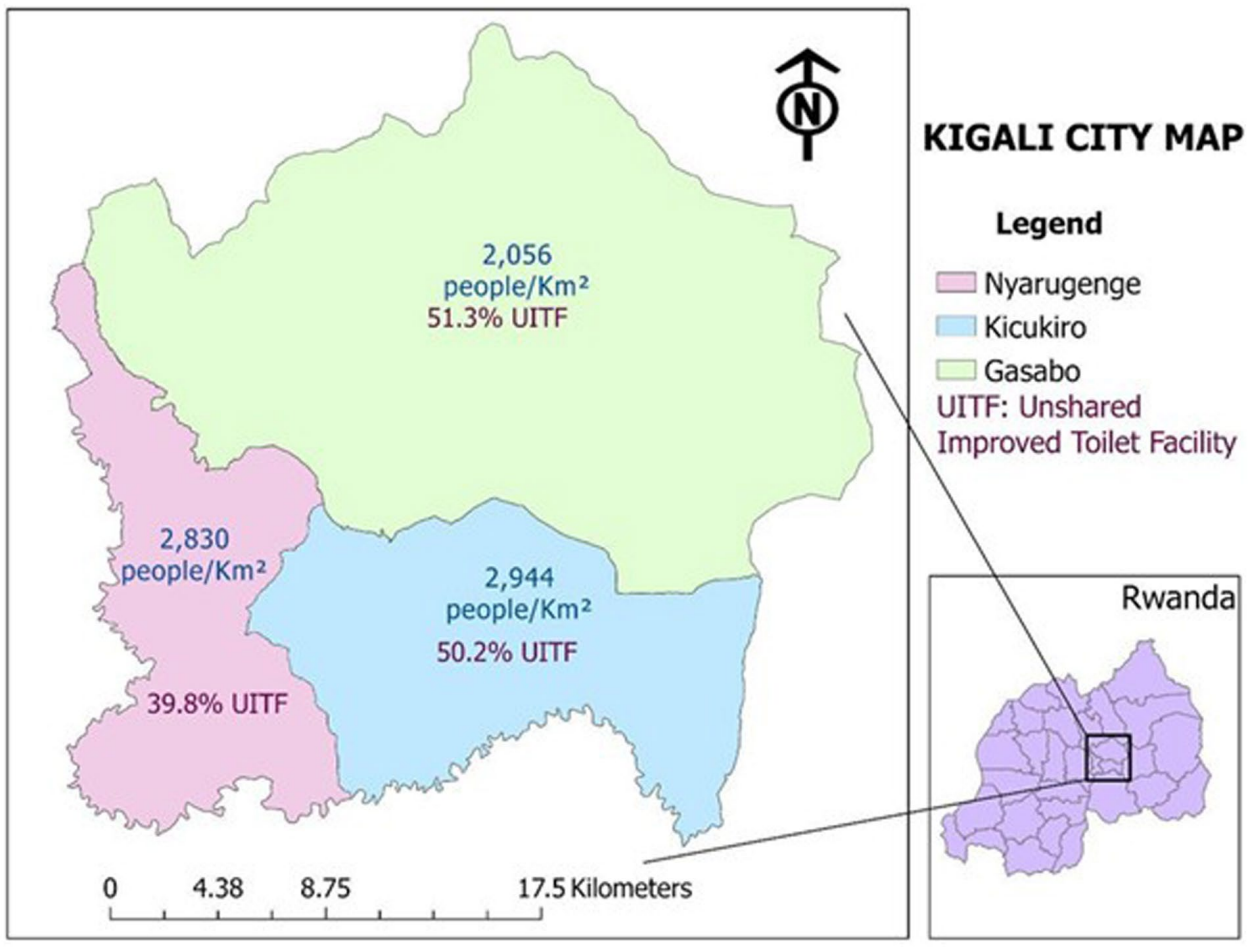
Study area showing the districts of Kigali, including population density and proportion of households with access to unshared improved toilet facilities.

The study was conducted in Kigali, the capital city of Rwanda. Kigali is characterized by rapid urbanization with 3% annual growth and the highest national population density of about 2,400 people/km^2^ (8). Geographically, the city is situated close to critical water bodies, including wetlands and rivers, namely the Nyabarongo River, Nyabugogo, Kiruhura, Nyandungu, and Masaka wetlands, and some streams and brooks, which are vulnerable to environmental contamination. Despite its urban profile, compared to other provinces, Kigali has the lowest proportion of households with access to unshared toilets, and the majority use OSSs, mainly pit latrines (8). Consequently, the city experiences challenges in urban fecal sludge management, potential fecal environmental contamination, and the need for enhanced sanitation services. The administrative boundaries of Rwanda are divided as follows, from the largest to the smallest: Provinces, Districts, Sectors, Cells, and Villages. Kigali City stands as a province, and has three districts: Kicukiro, Gasabo, and Nyarugenge (26). Figure 3 illustrates the three districts of Kigali, their respective density, and access to unshared improved toilet facilities. Gasabo District stands as the largest in size with the lowest population density, offering a diverse mix of settlements, and serving as the best focus area for this study due to its diverse population.

This study was conducted in Gasabo District, which was chosen as a focus area because it is the largest and most populous district in Kigali city, consisting of 15 sectors and featuring formal and informal settlements, wetlands, brooks, and hilly terrain. For this study, five sectors were purposively selected—Gisozi, Ndera, Rusororo, Kacyiru, and Kimironko. They were chosen based on the diversity in their settlements – including new planned settlements, old planned and unplanned settlements – and diverse population demographics, which provide a representative context of Kigali’s urban sanitation dynamics.

### 2.3 Data collection

#### 2.3.1 Sampling

A multistage cluster and random sampling design was used in one of Kigali’s three districts – Gasabo. In this district, five sectors were purposively selected, while random sampling was used for households in two to three cells (also randomly selected) in each sector. A cell is the administrative level below a sector, and the number of cells varies between sectors; in general, there are 4–7 cells per sector. Given that only one district was included, findings are not intended to be representative of all of Kigali.

To determine the sample size of households for the survey, the Equation 1 of Cochran (1977) was used:


$$n_{{}^{\circ}}=\frac{z^{2}*p*q}{e^{2}}$$ (1)

Where n_0_ is the sample size, z is the selected critical value for the desired confidence level, p is the estimated proportion of an attribute in the population, q = 1-p, and e is the desired level of precision.

The proportion was defined based on the recent Rwanda Population and Households Census (RPHC 5) of 2022, which showed that over 85% of households in the Gasabo district use pit latrines (8). For this study, a 95% confidence level, 5% precision, and an estimated proportion of 80% were used to improve precision and limit bias.

According to Cochran (1977), if the population is finite, the sample size calculated in (1) can be adjusted using Equation 2 as follows:


$$n=\frac{n_{{}^{\circ}}}{1+(\frac{n_{{}^{\circ}}-1}{N})}$$ (2)

Where n is the adjusted sample size, n_0_ is the sample size calculated in (1), and N is the population size.

Table 3 presents the calculated sample sizes for each sector – in total, 969 households were included in five sectors of Gasabo District. Included households were only surveyed if they had a pit latrine or a cesspit.

**Table 3:** Sample size adopted in this study.

Sector	Population size (*N*)	Calculated sample (*n*_0_)	Adjusted sample (*n*)
Gisozi	22,899	195.92	194
Ndera	28,286	195.92	195
Rusororo	16,650	195.92	194
Kairu	8,918	195.92	192
Kimironko	17,612	195.92	194
Total			969

#### 2.3.2 Ethical consideration and quality assurance

Ethical clearance was sought and obtained from the University of Rwanda Research and Postgraduate Studies Committee (RPSC) – no protocol number was issued. The authorization to conduct the household survey was granted by the Kigali City Authorities. After receiving an explanation of the research purpose and verifying Kigali city’s authorization, household members were requested by enumerators to participate in the survey. They were also informed that their information will be used, safeguarded, and kept confidential. Those who agreed to participate in the survey signed a consent form.

All collected data were securely stored and treated with strict confidentiality. Access to the data was restricted to authorized members of the research team. After data analysis, findings were reported in aggregate form, ensuring no identifiable information was disclosed. All this data safeguard and confidentiality were done in adherence to UR-RPSC guidelines.

The survey was conducted from December 2023 to February 2024 and was administered using mWater, a digital platform for WASH data management, used to measure impact, access to services, and record data.

During 2 days, enumerators were trained in using mWater, and testing sessions were conducted to further understand the characteristics detailed in Table 1 before the deployment of the survey. Additionally, before data collection, enumerators went through a comprehensive training covering the use of the tool, ethical and safety considerations during fieldwork, procedures for obtaining informed consent, and principles of confidentiality and transparency. After deployment, adjustment of the tool during field work was done, adapting questions and characteristics to the realities of the field. Enumerators were supervised by a Team Leader (TL), a public health and mWater specialist, under the overall direction of the Principal Investigator (PI). The training sessions for enumerators were conducted jointly by the TL and the PI to ensure methodological consistency and ethical consideration. The PI convened regular check-in meetings with three co-researchers (PhD supervisors) for brainstorming and providing updates before, during, and after data collection. This practice ensured continuous oversight and integration of supervisory feedback into data collection. During data collection, the PI and the TL maintained active interaction with enumerators. This consultation enabled real-time problem-solving and methodological adjustment.

### 2.4 Data analysis

Data were first cleaned to remove non-qualifying responses and were then analyzed to assess the level of compliance of pit latrines and cesspits with RS ISO 24521, identify associations with compliance, and examine the relationships between characteristics of selected objectives and key selected technical and demographic characteristics.

Statistical analyses were done using IBM SPSS 20.0. Four levels of compliance were used – Full Compliance, Moderate Compliance, Low Compliance, and Non-Compliance (see Table 2). The compliance scoring framework in Table 2 was used to calculate the frequencies of pit latrines and cesspits meeting the four compliance levels; however, for a household to be considered as fully compliant with a standard objective, all characteristics assessed under each objective were required to be met. All characteristics within each objective were treated as equally weighted, consistent with the structure of RS ISO 24521, which does not assign differential priority to specific requirements. However, this approach remains susceptible to bias, given that certain data are self-reported and others originate from direct observations.

A Generalized Linear Mixed Model (GLMM) with binomial probability distribution and logit link function was used to examine the association between demographic characteristics (set as fixed effects) and full compliance with the four selected standard objectives (set as outcomes). The demographic characteristics used as fixed effects in the GLMM are detailed in Table 1, and include Wealth of household, Residential type, Type of settlement, House tenure, Presence of piped water, and Age of the pit. The outcome variables were binary, 0 = Not complying with all characteristics, 1 = Full compliance. The model estimated the probability of compliance, and parameters were estimated using Maximum Likelihood methods as implemented in IBM SPSS 20.0. Odds were obtained by exponentiating the fixed effect coefficients. Random effects and intercepts were specified at the sector level to account for clustering, while sample weights were calculated and applied at the same level to ensure representativeness of the estimates within the five sampled sectors of Gasabo District. Although other compliance-level analysis approaches were feasible, Full Compliance was prioritized because it is directly aligned with the safely managed sanitation service indicator under SDG 6.2. This level is highly unrepresentative and remains significantly underachieved in Kigali (2, 27, 28). For these reasons, this underexplored dimension was deliberately examined to contribute to a more nuanced understanding of sanitation services disparities.

To assess bivariate associations between technical and demographic characteristics and specific characteristics within each standard objective, Rao-Scott adjusted Chi-Square tests were used to account for the sampling design, where clustering was considered at the sector level. Technical and demographic characteristics were selected based on the research team’s assessment of their importance for promoting safe sanitation practices.

For both Chi-Square and GLMM, associations were considered significant if the *p* ≤ 0.05. Direction of associations was extracted from the sign of the model coefficient, and Odds ratios (OR) were used to measure the strength of the association, where OR>1 increases the odds of the outcome. Moreover, 95% Confidence Interval (CI) was used to evaluate the precision of the estimate, where a narrow CI represents a more precise estimate. For both statistical models, sampling weights were computed at the sector level and applied to all households within each sampled sector in Gasabo District. This analytical approach was chosen to align with the objective of the study to identify factors associated with households achieving full compliance with selected standard objectives. Sample weight was calculated using Equation 3 of Yansaneh (29):


$$W_{i}=\frac{1}{P_{\mathrm{ij}}}\text{ where }P_{\mathrm{ij}}=P_{i}*P_{j}$$ (3)

Where *W_i_* represents the weight and *P_i_* probability of the sample being selected into the sample.

In IBM SPSS 20.0, GLMM with binomial probability distribution and logit link function was implemented using the GENLINMIXED (path: Mixed Models-Generalized Linear) procedure, while Rao-Scott adjusted Chi-square was implemented through the Complex Sample Crosstabs procedure.

A key limitation in the data analysis and interpretation in this study is the reliance on self-reported and direct observation measures of sanitation compliance characteristics. Self-reported characteristics such as income, satisfaction, knowledge of regulations, and others (Table 1) may be affected by recall and social desirability biases. On the other hand, characteristics directly observed by enumerators, such as cleanliness or facility condition, and other listed in Table 1, may be affected by observer misclassification, lack of inter-rater reliability, or missed observation. Despite quality checks, the respondents may have over- or under-reported information to align with perceived expectations, while enumerators may have overlooked some characteristics during inspection. Moreover, the rarity of some compliance outcomes (e.g., very low levels of full compliance) can lead to instability in the statistical tests. Therefore, the data analysis and interpretation were conducted considering potential misclassification of outcomes and the reliability of statistical results.

## 3 Results

### 3.1 Demographics of sanitation facility usage

**Figure 4:**
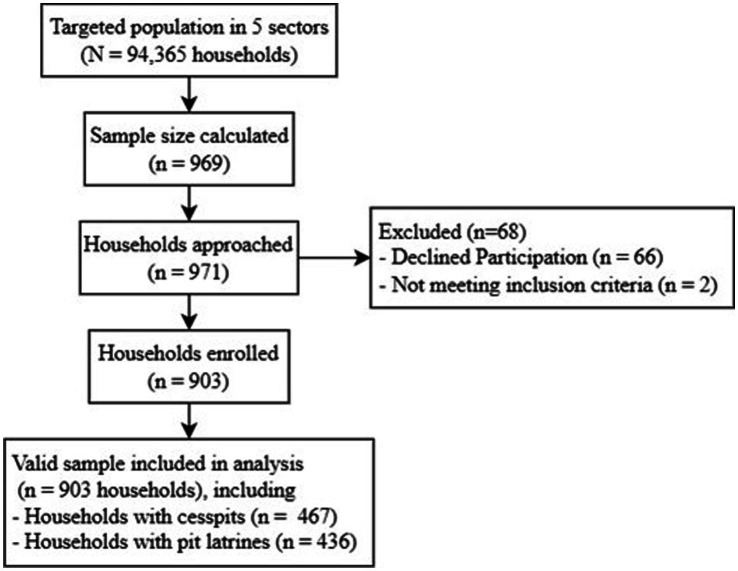
Participant flow diagram from sampling to analysis.

The survey achieved a response rate of 93%, with 903 valid responses included in the analysis. Of these, 436 were households using pit latrines (48%), and 467 were households using cesspits (52%). A total of 66 households declined participation in the survey, and 2 responses were excluded because they did not meet the inclusion criteria, as illustrated in Figure 4. The results show that 85% of pit latrines were used by low and middle-income households, and 91% of cesspits were used by middle and high-income households (Table 4). For both types of pits, middle-income households were predominant. However, low-income households are unequally represented among cesspit users, where these facilities are predominantly used by middle and high-income households, 57 and 37%, respectively. Sharing pits was more common among pit latrine users (62.6%) than among cesspit users (7.5%). The average number of households sharing one pit latrine was 3.4.

**Table 4:** Demographic characteristics related to the sanitation facilities.

Demographic characteristics	Pit latrines (%)	Cesspits (%)
Wealth of households		
Very low income	11	1
Low income	40	8
Middle income	45	57
High income	4	34
Residential type		
Compound with many households	62	31
Single house	38	69
Type of settlement		
Old settlement	87	56
New settlement	13	44
House tenure		
Owning	44	68
Renting	56	32
Presence of piped water		
Yes	77	96
No	23	4
Age of pit		
Below 5 years	18	22
Between 5 and 10 years	35	50
Over 10 years	47	28

**Figure 5:**
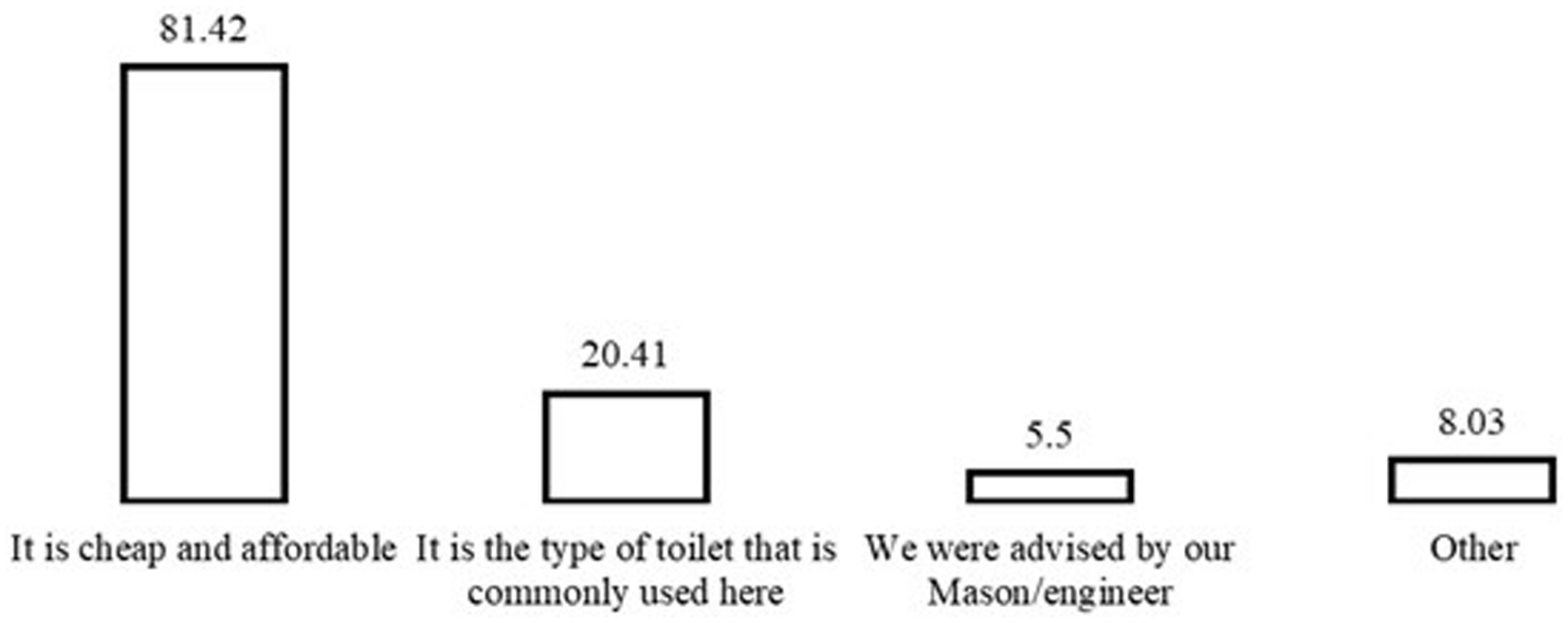
Reasons households opted for pit latrines as sanitation facilities (%).

**Figure 6:**
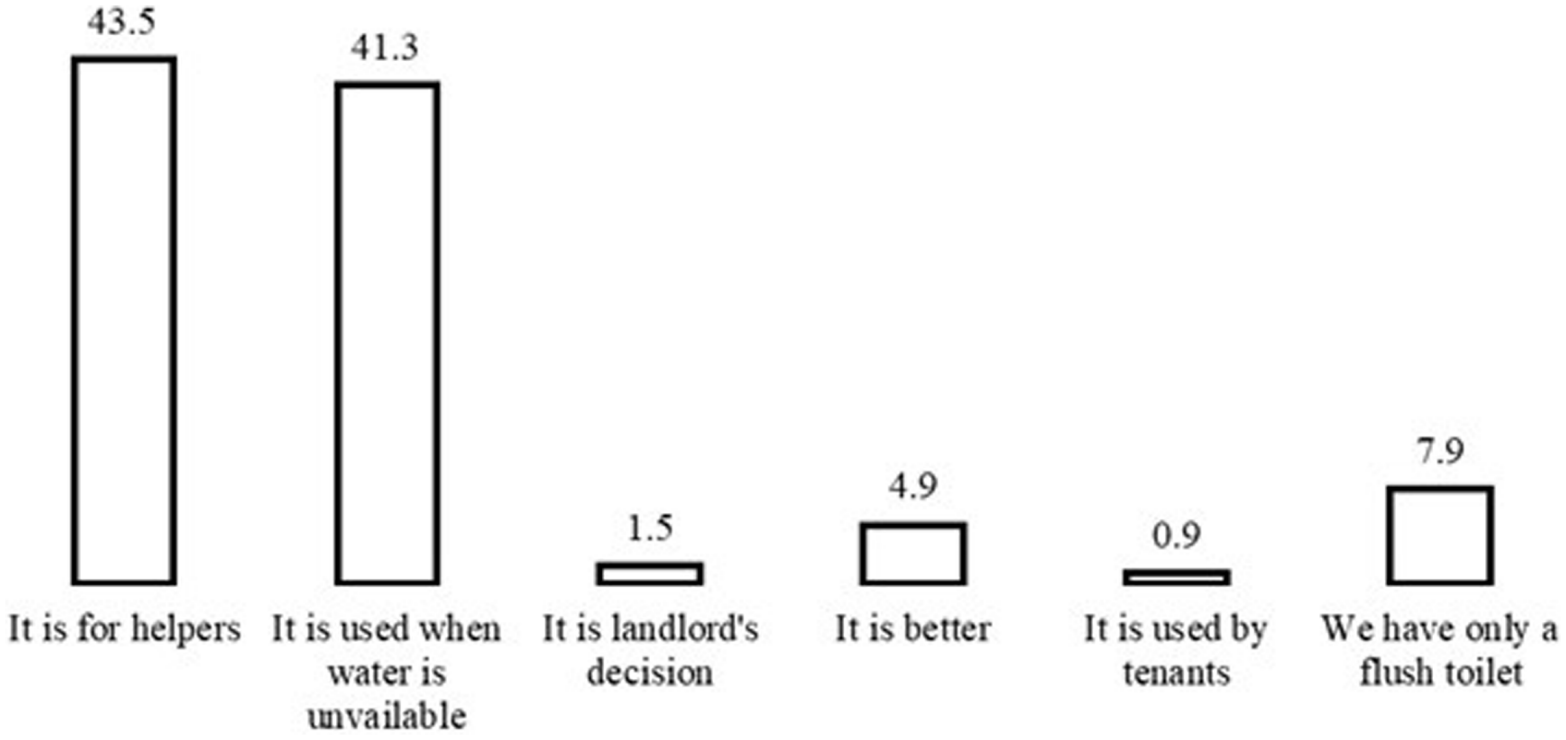
Reasons for households having both flushing toilets and pit latrines (%).

To further improve the understanding of household features influencing the type of sanitation services chosen by households, Figures 5, 6 present household-reported motivations underlying the choice of sanitation facilities. Figure 5 shows that the majority of pit latrine users (81.4%) indicated that they have chosen this type of facility because it is cheap and commonly used, while 20.4% said they adopted it because it is locally commonly used. Other 5.5% claimed that they were advised by their technician. During the survey, it was noted that the majority of households using cesspits also had a pit latrine. As illustrated in Figure 6, two reported motivations for this practice were that pit latrines are mainly for domestic workers (43.5%) and serve as a contingency option when water for flushing is unavailable (41.3%). Others claimed it was a decision made by their tenants, or the facility was intended to be used by tenants. These results underscore the importance of water supply and behavior (especially when the facility is intended for helpers and tenants) in influencing the choice of a sanitation facility.

### 3.2 Compliance with RS ISO 24521

#### 3.2.1 All standard objectives

**Figure 7:**
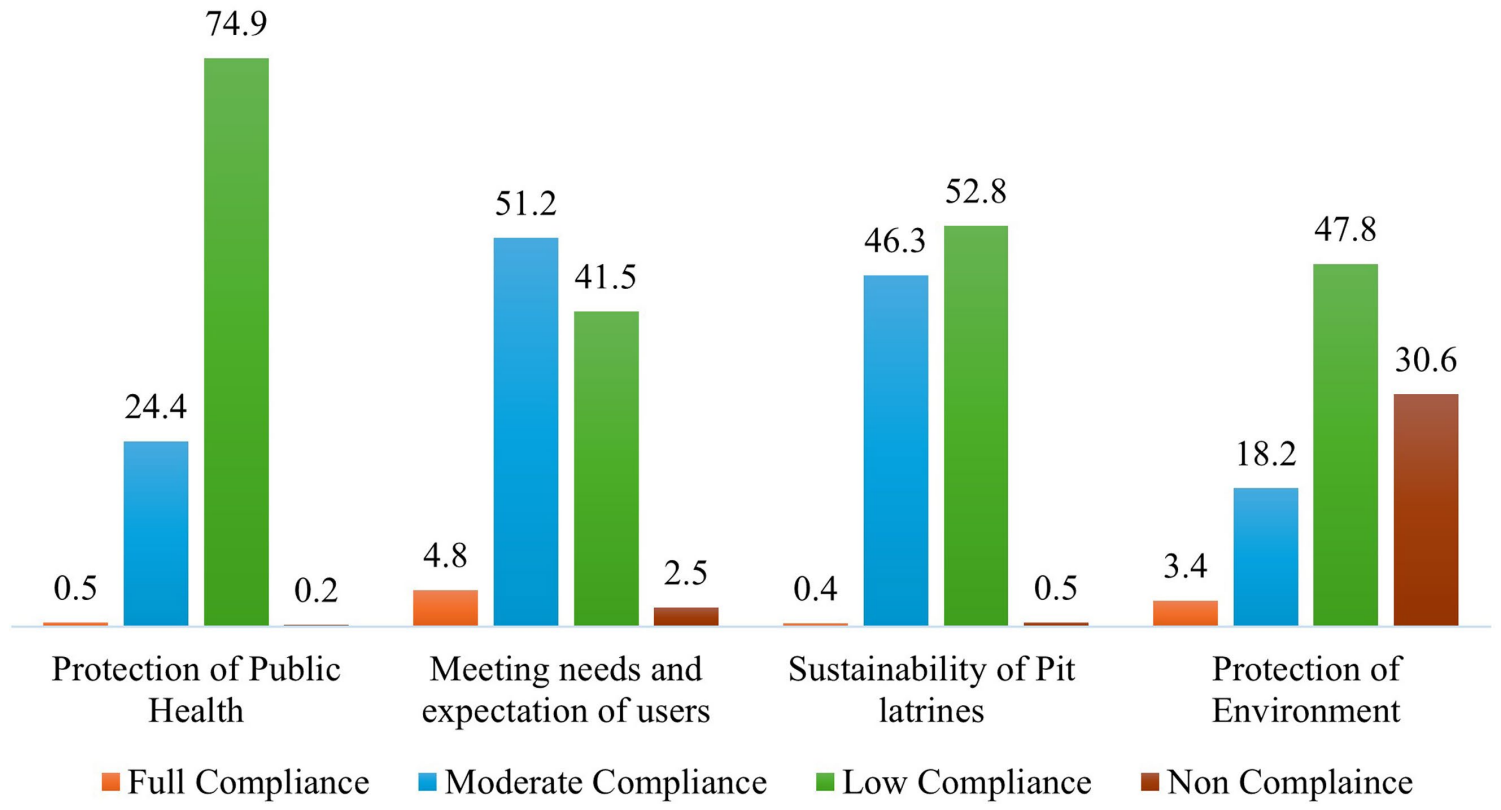
Pit latrines compliance levels in % with selected objectives of RS24521.

**Figure 8:**
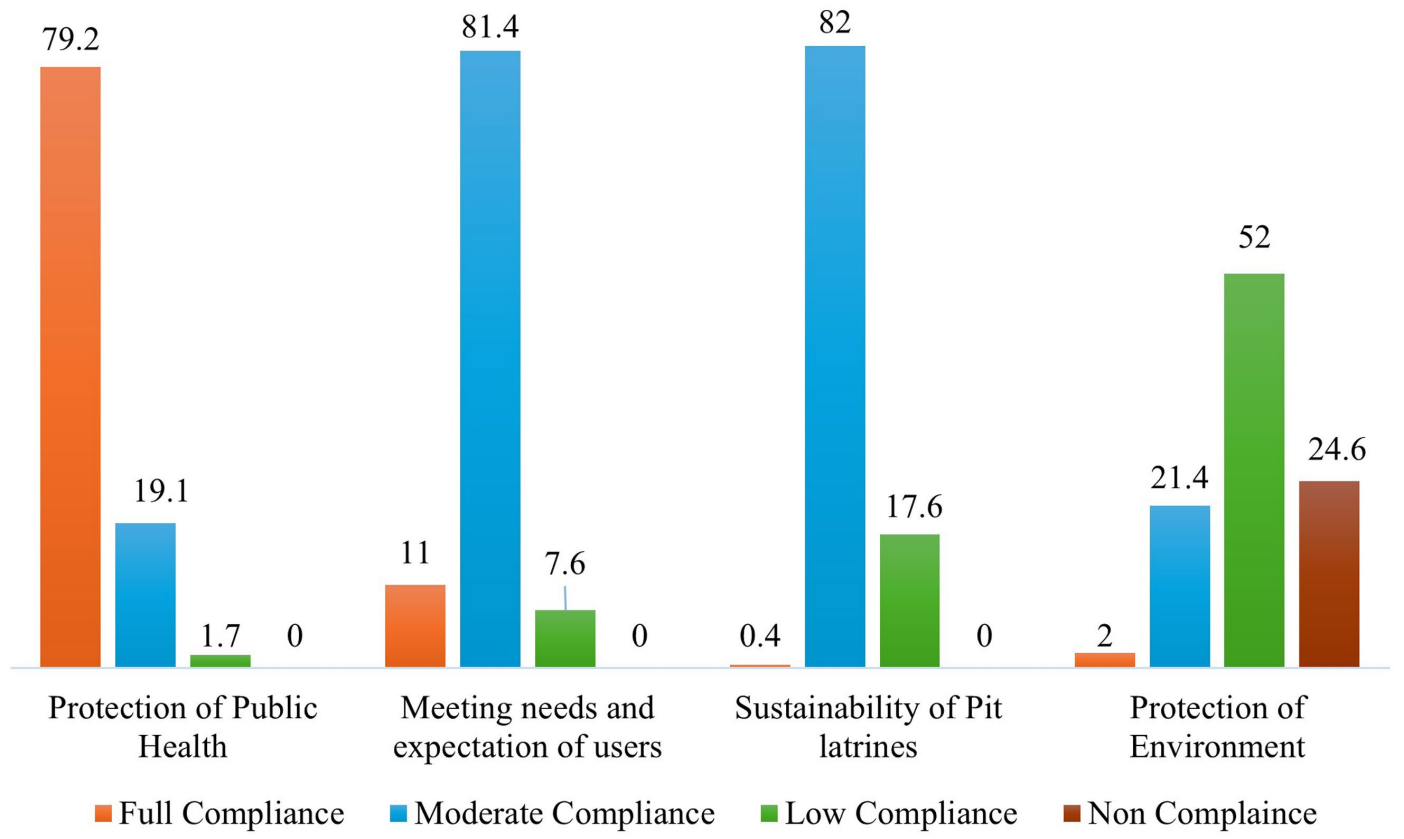
Cesspits compliance levels in % with selected objectives of RS24521.

**Figure 9:**
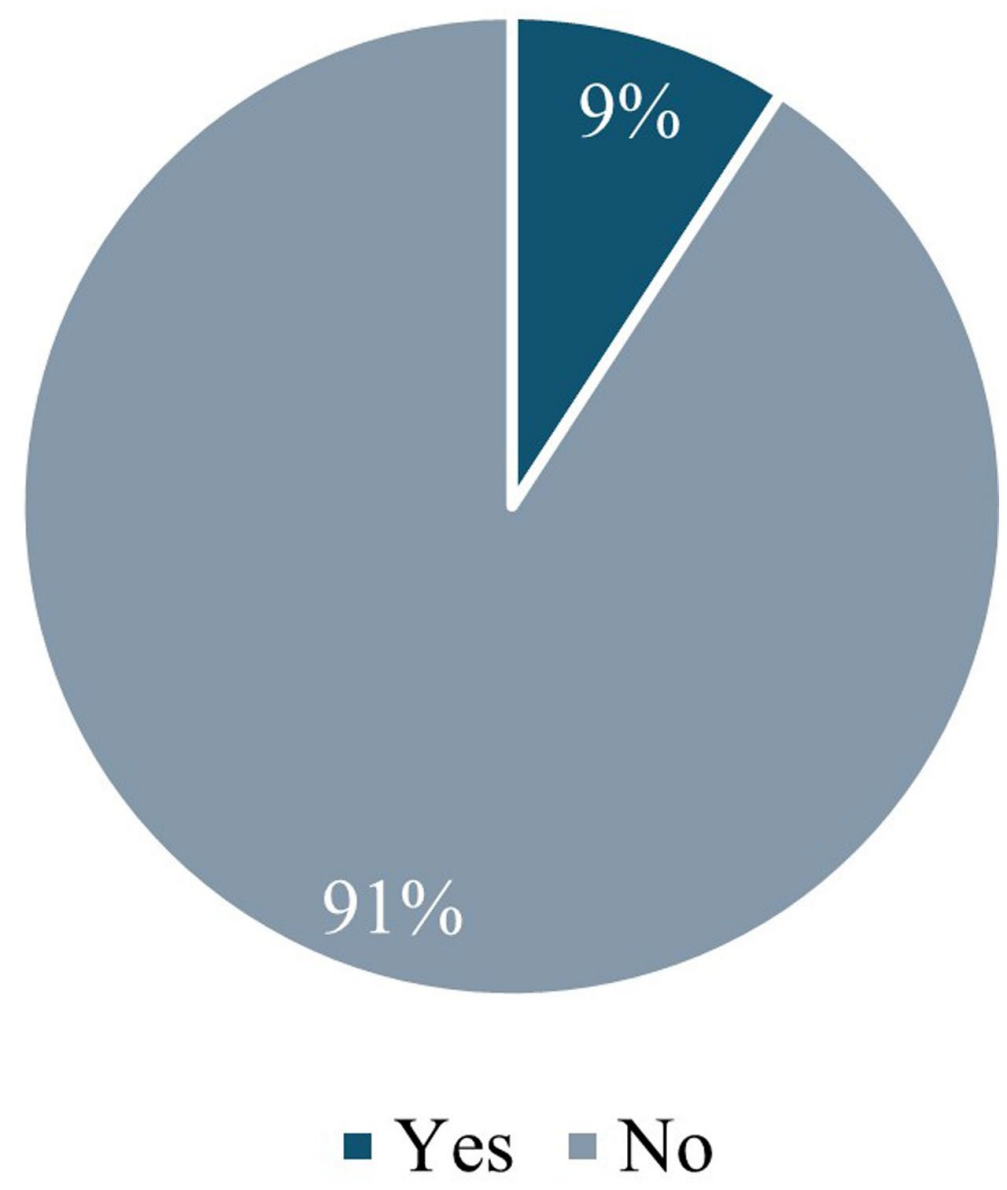
Prevalence of handwashing facilities near pit latrines.

The results on compliance levels with the four selected objectives of the standard are provided in Table 5, which illustrates how compliance of surveyed facilities with RS ISO 24521 is generally low. Cesspits demonstrated a high score of Moderate Compliance with Meeting Needs and Expectations of Users as well as with Sustainability (Table 5 and Figure 7). In contrast, pit latrines exhibited scores distributed across moderate and low compliance for these two objectives (Table 5 and Figure 8). Regarding the objective of Protection of the Environment, both types of facilities have nearly equal low and non-compliance scores. This shows that the full compliance is very low for all objectives except the Protection of Public Health for cesspits. For example, very few of the investigated pits fully comply with Sustainability, suggesting that most pit latrines and cesspits may not be sustainable. Similarly, full compliance of pit latrines with Meeting Users’ Needs and Expectations is low, mainly due to a high rate of dissatisfaction with the facility and of sharing it with other households. While cesspits have a high compliance with Public Health Protection, pit latrines are far below in this regard. However, the comparison between pit latrines and cesspits remains partial because they have different number of characteristics, especially for the Protection of Public Health and Sustainability objectives (Figure 9).

**Table 5:** Compliance frequencies (%) for selected objectives of RS ISO 24521 and related characteristics in surveyed pit latrines (PL) and cesspits (CP).

Standard objective	Characteristic	Compliance frequency – characteristic (%)		Compliance frequency - standard objective (%)							
				Pit latrines				Cesspits			
		PL	CP	Full	Med.	Low	None	Full	Med.	Low	None
(1) Protection of Public Health	Pit has no evidence of leaking into the surrounding environment	58.9	–	0.5	24.4	74.9	0.2	79.2	19.1	1.7	0
	Interface (only for pit latrines) and pit surface are clean	10.8	95.3								
	No presence of flies	49.7	94.2								
	Pit’s smell is not bad	27.1	93.1								
	Presence of handwashing	9.2	–								
	Piped water is available in the compound	–	95.9								
	Pit has no gas stinging in the eyes	73.6	92.7								
(2) Meeting Needs and Expectations of Users	Facility provides security to intended users	54.6	89.5	4.8	51.2	41.5	2.5	11.0	81.4	7.6	0
	Facility not shared with other households	37.4	92.5								
	Users’ satisfaction with the facility	25.5	76.4								
	Pit is not a danger to other houses (leaking, risks of collapse)	74.1	93.6								
	Affordable	81.4	23								
(3) Sustainability	Reinforced concrete slab is used	70.1	91	0.4	46.3	52.8	0.5	0.4	82	17.6	0
	Lined pit is used	8.1	21.5								
	Superstructure in good condition	64.7	–								
	Slab and pit in good condition	62	68								
	Pit has been emptied at least once or it is new	13.5	13.1								
	Rainwater does not flow into pit	80.5	85.7								
	Only excreta (for pit latrines) and black water (for cesspits) are discharged into the pit	72	78.8								
	Pit cover has an opening for emptying services	10.3	36.4								
	Residence is accessible by a road at least 3 m wide	74.3	87.2								
	Pit can be easily accessed for emptying services	36.6	53.7								
	Knowledge of regulations about facility construction	6.7	37.3								
(4) Protection of the Environment	Pits with a depth of <10 m	17.2	11.1	3.4	18.2	47.8	30.6	2	21.4	52	24.6
	Groundwater or moist soil was not reached while digging the pit	49.3	66.7								
	Authorities inspect the facility	28	17.3								
Overall				0	39.8	60.2	0	0	94.4	5.6	0

Tables 6, 7 present the estimated fixed-effect coefficients, *p*-values, 95% Confidence Intervals (CI), and Random effects covariance derived from the GLMM with random intercepts at the sector level, which were utilized to examine the probable extent to which demographic characteristics may be associated with compliance with selected objectives of the standard. The results provide insight into the relative strength and direction of association between each demographic characteristic and the likelihood of compliance.

**Table 6:** Analysis of associations between demographic characteristics and compliance with standard objectives of RS ISO 24521 – for pit latrines by the generalized linear mixed model (GLMM) with binomial probability distribution and logit link function (reference category: full compliance).

Standard objective		Protection of public health				Meeting needs and expectations of users				Sustainability				Protection of the environment			
		Random effect covariance: NA				Random effect covariance: 3.475				Random effect covariance: NA				Random effect covariance: 6.620			
Demography ↓		*β*	*p*	Odds	95% CI	*β*	*p*	Odds	95% CI	*β*	*p*	Odds	95% CI	*β*	*p*	Odds	95%CI
Characteristics	Categories																
Wealth/Income	Very Low Income	–	–	–	–	1.58*	0.00	4.86	(1.42; 1.75)	–	–	–	–	2.21*	0.00	9.10	(1.94; 2.47)
	Low Income	–	–	–	–	0.87*	0.00	2.38	(0.76; 0.98)	–	–	–	–	2.37*	0.00	10.68	(2.18; 2.55)
	Middle Income	–	–	–	–	0.26*	0.00	1.30	(0.17; 0.36)	–	–	–	–	2.28*	0.00	9.76	(2.10; 2.46)
	High Income (REF)																
Residential type	Compound with many tenants	–	–	–	–	1.58*	0.00	4.85	(1.52; 1.64)	–	–	–	–	13.86	0.47	1,044,448	(−23.54; 51.25)
	Single–family house (REF)																
Type of settlement	New settlement	–	–	–	–	12.09	0.24	178,617	(−8.30; 32.49)	–	–	–	–	−0.84*	0.00	0.43	(−0.99; −0.68)
	Old settlement (REF)																
House tenure	Renting	–	–	–	–	1.00*	0.00	2.72	(0.94; 1.06)	–	–	–	–	−0.76*	0.00	0.47	(−0.89; −0.62)
	Owning (REF)																
Presence of piped water in the compound	No	–	–	–	–	−0.32*	0.00	0.72	(−0.40; −0.25)	–	–	–	–	−0.85*	0.00	0.43	(−1.00; −0.70)
	Yes (REF)																
Age of the facility	Less than 10 years	–	–	–	–	−0.01	0.86	1.00	(−0.06; 0.05)	–	–	–	–	−13.20	0.53	0.00	(−54.01; 27.62)
	Over 10 years (REF)																

*β*: Fixed effect coefficient, bold *β* marked with * indicates a significant estimated means contrast, with the least adjusted significant level set at 0.05, *p*: Significance (*p* < 0.05 is significant), CI: Confidence Interval.

**Table 7:** Analysis of associations between demographic characteristics and compliance with standard objectives of RS ISO 24521—for cesspits by the Generalized Linear Mixed Model (GLMM) with binomial probability distribution and logit link function (reference category: full compliance).

Standard objective →		Protection of public health				Meeting needs and expectations of users				Sustainability				Protection of the environment			
		Random effect covariance: 0.124				Random effect covariance: 0.260				Random effect covariance: NA				Random effect covariance: 4.956			
Demography ↓		*β*	*p*	Odds	95% CI	*β*	*p*	Odds	95% CI	*β*	*p*	Odds	95% CI	*β*	*p*	Odds	95% CI
Characteristics	Categories																
Wealth/Income	Very Low Income	1.71	0.00	5.55	(1.596; 1.831)	−1.24*	0.00	0.29	(−1.39; −1.09)	–	–	–	–	−2.51*	0.00	0.08	(−2.67; −2.35)
	Low Income	1.13	0.00	3.10	(1.075; 1.188)	0.73*	0.00	2.09	(0.67; 0.81)	–	–	–	–	−1.43*	0.00	0.24	(−1.58; −1.29)
	Middle Income	0.50	0.00	1.65	(0.465; 0.545)	1.60*	0.00	4.96	(1.56; 1.65)	–	–	–	–	1.13*	0.00	3.12	(1.03; 1.25)
	High Income (REF)																
Residential type	Compound with many tenants	0.74	0.00	2.11	(0.716; 0.778)	−1.00*	0.00	0.37	(−1.04; −0.96)	–	–	–	–	−0.08	0.06	0.91	(−0.18; 0.00)
	Single–family house (REF)																
Type of settlement	New settlement	−0.40	0.00	0.66	(−0.439; −0.366)	−0.47*	0.00	0.62	(−0.52; −0.43)	–	–	–	–	0.63*	0.00	1.89	(0.53; 0.74)
	Old settlement (REF)																
House tenure	Renting	0.59	0.00	1.82	(0.567; 0.631)	0.37*	0.00	1.46	(−0.42; −0.33)	–	–	–	–	−1.13*	0.00	0.32	(−1.23; −1.04)
	Owning (REF)																
Presence of piped water in the compound	No	12.29*	0.00	219,476	(−0.813; −0.747)	−0.98*	0.00	0.37	(−1.05; −0.92)	–	–	–	–	12.63	0.80	308,044	(−87.64; 112.92)
	Yes (REF)																
Age of the facility	Less than 10 years	−0.78	0.00	0.45	(−0.813; −0.747)	1.28*	0.00	3.61	(1.24; 1.33)	–	–	–	–	−12.76	0.60	0.00	(−61.62; 36.09)
	Over 10 years (REF)																

*β*: Fixed effect coefficient, bold *β* marked with * indicates a significant estimated means contrast, with the least adjusted significant level set at 0.05, *p*: Significance (*p* < 0.05 is significant), CI: Confidence Interval.

For pit latrines, wealth, residential type, house tenure, and presence of piped water were significantly associated with compliance with the standard objective, Meeting Needs and Expectations of Users. Similarly, wealth, type of settlement, house tenure, and presence of piped water demonstrated a significant association with compliance with the standard objective Protection of Environment (Table 6). For cesspits, all investigated demographic characteristics had significant associations with compliance with the standard objectives Protection of Public Health, and Meeting Needs and Expectations of Users. Only wealth, type of settlement, and house tenure exhibited a significant association with compliance with the Protection of Environment (Table 7).

For both pit latrines and cesspits, attempts to fit the GLMM with the standard objective Sustainability were unsuccessful due to the rarity of full compliance (<1%). A similar scenario was observed for the standard objective Protection of Public Health for pit latrines (with full compliance of <1%). Given that fewer than 1% full compliant facilities were observed in these scenarios, the lack of variation precluded reliable parameter estimation. The rarity of these full compliance cases demonstrates that the overwhelming majority of samples did not fully comply, suggesting a high prevalence of unsustainable pit latrines and cesspits, and pit latrines that do not protect public health across all demographics represented in the samples.

#### 3.2.2 Protection of public health

For the standard objective, Protection of Public Health, the prevalence of non-compliant characteristics among pit latrines was notably high. Specifically, 89.2% of facilities were characterized as unhygienic, 90.8% lacked a handwashing station, and 72.9% exhibited a noticeable bad smell. Collectively, these deficiencies substantially undermined the compliance with this objective, resulting in an overall full compliance score of 0.5%. In contrast to pit latrines, the overall full compliance of cesspits with the Protection of Public Health is far higher, with a score of 79.4%. This is mainly because all investigated characteristics under this standard objective perform well for cesspits (above 90%). This may be linked to the fact that, in general, cesspits are located away from the user interface (flushing toilets) and entirely covered by reinforced concrete slabs.

In the GLMM, among cesspit users, lower socioeconomic status was strongly associated with reduced likelihood of full compliance (Table 7). Compared with high-income, those in the very low-, low-, and middle-income categories had significantly higher odds of non-compliance, indicating clear variation in the socioeconomic dimension. Households living in compounds with many other households were more than twice as likely to be non-compliant compared to those in single-family houses. In contrast, cesspits in new settlements and having newer cesspits were associated with lower odds of non-compliance compared to cesspits in old settlements and households with older cesspits, suggesting that living in a compound with many households and in an old settlement may increase barriers to having a cesspit that protects public health. Similarly, not owning a house was also associated with increased odds of non-compliance. Younger cesspits are less likely to be non-compliant compared to those aged over 10 years. Lack of piped water had the highest probability of non-compliance. The odds were higher compared to households with piped water. The random effects variance suggested weak positive clustering across sectors. This finding suggests that full compliance with the standard objective Protection of Public Health for cesspits may be shaped by investigated demographic characteristics.

Table 8 presents the results of the Rao-Scott Chi-Square analysis of the association between demographic and key technical characteristics and the characteristics of the standard objective Protection of Public Health. The results show that having a clean cesspit was significantly associated with residential type, settlement type, and the age of the facility. Shared facilities were slightly cleaner than non-shared, suggesting that communal use of cesspits encourages hygienic practices; the opposite was found for pit latrines. However, as income increases, the likelihood of maintaining a cleaner pit also rose, suggesting that low-income households may face barriers in maintaining hygienic cesspits. Pit latrines that were newly constructed or frequently emptied demonstrated a higher likelihood of being maintained in a hygienic condition compared to those that were never emptied. This pattern suggests that emptying, as a routine maintenance practice, may be positively linked to the promotion of hygiene practices for the facility. However, bad smell in pit latrines increased with the frequency of emptying events, suggesting a probable imbalance of physicochemical processes. Clean pit latrines were significantly associated with house tenure, where the likelihood of having a clean pit latrine increased with owning the house.

**Table 8:** Rao-Scott chi-square analysis of associations between protection of public health characteristics and key technical and demographic characteristics.

Technical and demographic → Public health protection ↓			Sharing facility	Pit conditions	Depth of the pit	Emptying frequency	Lining status	Wealth	House tenure	Residential type	Settlement type	Presence of piped water	Age of the pit
No evidence of leaking CP	CP	Adjusted F	–	–	–	–	–	–	–	–	–	–	–
		Odds ratio	–	–	–	–	–	–	–	–	–	–	–
	PL	Adjusted F	0.06	0.22	5.87	0.21	4.45	9.56*	0.95	0.54	67.90*	5.70	1.56
		Odds ratio	(0.76, 1.38)	(0.43, 1.80)	(0.44, 1.05)	(0.54, 1.97)	(0.60, 16.79)	(0.57, 0.97)	(0.66, 2.36)	(0.58, 2.54)	(0.23, 0.50)	(0.35, 1.08)	(0.77, 1.95)
Interface (only for PL) and surface are clean CP	CP	Adjusted F	5.31	0.05	0.08	0.91	0.02	3.92	4.64	8.93*	15.61*	2.02	10.43*
		Odds ratio	(0.89, 16.43)	(0.25, 4.96)	(0.10, 15.24)	(0.08, 117.2)	(0.35, 3.21)	(0.66, 33.15)	(0.70, 14.86)	(1.08, 7.17)	(0.01, 0.86)	(0.38, 38.99)	(0.03, 0.82)
	PL	Adjusted F	0.03	0.84	0.23	6.65	6.27	3.06	7.86*	0.48	1.50	2.13	6.59
		Odds ratio	(0.58, 1.84)	(0.32, 1.75)	(0.31, 2.26)	(0.95, 5.11)	(0.97, 9.85)	(0.73, 3.87)	(1.00, 2.16)	(0.43, 4.00)	(0.12, 2.14)	(0.77, 2.18)	(0.22, 1.08)
No flies	CP	Adjusted F	9.37*	0.51	3.40	2.60	0.83	25.62*	0.11	3.21	0.03	0.01	10.77*
		Odds ratio	(1.13, 4.41)	(0.43, 4.13)	(0.32, 38.17)	(0.36, 25.10)	(0.38, 6.29)	(1.80, 5.91)	(0.23, 3.09)	(0.53, 18.66)	(0.47, 1.92)	(0.02, 33.06)	(0.11, 0.82)
	PL	Adjusted F	0.04	7.16*	32.85*	1.45	0.54	0.68	1.32	8.93*	17.58*	0.23	4.10
		Odds ratio	(0.43, 2.04)	(0.47, 1.01)	(0.43, 0.75)	(0.78, 1.09)	(0.61, 2.30)	(0.69, 1.95)	(0.81, 1.08)	(1.01, 1.46)	(0.19, 0.73)	(0.52, 1.57)	(0.31, 1.20)
The pit smells not bad	CP	Adjusted F	0.96	0.39	0.06	72.24*	0.09	3.61	0.38	0.31	2.66	6.09	3.79
		Odds ratio	(0.38, 8.33)	(0.08, 4.94)	(0.17, 8.28)	(3.24, 18.36)	(0.37, 2.18)	(0.73, 7.67)	(0.27, 7.81)	(0.44, 3.43)	(0.18, 1.57)	(0.96, 2.76)	(0.04, 1.70)
	PL	Adjusted F	4.81	6.87*	0.22	4.72	0.62	2.03	2.28	42.96*	2.44	30.12*	1.61
		Odds ratio	(0.47, 1.08)	(0.98, 1.71)	(0.47, 2.89)	(0.30, 1.14)	(0.14, 2.91)	(0.85, 1.65)	(0.35, 1.35)	(0.51, 0.76)	(0.79, 2.34)	(1.35, 2.48)	(0.71, 2.50)
Presence of handwashing for PL	CP	Adjusted F	–	–	–	–	–	–	–	–	–	–	–
		Odds ratio	–	–	–	–	–	–	–	–	–	–	–
	PL	Adjusted F	0.14	0.19	13.18*	2.36	2.35	0.01	1.91	2.79	1.31	1.45	0.00
		Odds ratio	(0.45, 2.83)	(0.23, 2.88)	(0.14, 0.87)	(0.69, 3.67)	(0.50, 15.52)	(0.29, 2.99)	(0.39, 1.36)	(0.70, 4.18)	(0.23, 1.78)	(0.30, 1.58)	(0.20, 4.37)
Presence of piped water	CP	Adjusted F	0.22	0.59	0.01	4.92	0.05	1.48	0.38	1.55	0.41	–	1.21
		Odds ratio	(0.02, 15.85)	(0.05, 5.52)	(0.13, 6.24)	(0.51, 35, 14)	(0.21, 6.20)	(0.36, 17.14)	(0.27, 7.88)	(0.36, 14.92)	(0.24, 2.39)	–	(0.21, 1.93)
	PL	Adjusted F	–	–	–	–	–	–	–	–	–	–	–
		Odds ratio	–	–	–	–	–	–	–	–	–	–	–
No gas stinging in eyes	CP	Adjusted F	4.46	1.12	0.00	0.00	2.59	6.15	2.94	6.11	22.82*	3.44	0.03
		Odds ratio	(0.79, 12.82)	(0.43, 6.57)	(0.18, 5.36)	(0.19, 4.97)	(0.16, 1.56)	(0.97, 14.48)	(0.69, 5.07)	(0.87, 10.21)	(0.10, 0.61)	(0.00, 0.00)	(0.34, 3.15)
	PL	Adjusted F	4.46	12.14*	0.10	1.88	6.45	61.06*	13.45*	9.08*	0.00	311.98*	5.18
		Odds ratio	(0.86, 2.85)	(1.13, 2.96)	(0.45, 1.87)	(0.72, 2.56)	(0.94, 2.39)	(1.97, 4.37)	(1.07, 1.72)	(1.03, 2.65)	(0.42, 2.28)	(3.27, 5.09)	(0.60, 1.05)

Adjusted F: Rao-Scott Chi-square adjusted F statistic x2, Adjusted *F* values market in bold with * have *p* < 0.05.

The absence of flies around the facility was significantly associated with sharing the facility, wealth, and age of the pit for cesspits. Flies were more likely to be present in shared and older cesspits, and the presence of flies in middle and high-income households suggests improper hygienic practices. On the other hand, the absence of flies was significantly associated with pit conditions, pit depth, and residential and settlement types for pit latrines. The prevalence of flies around the pit latrine was more likely to happen in older settlements, shared compounds, deeper pits, and pits in good conditions, suggesting limited hygiene behaviors and structural associations with vector control in these settings.

Additionally, the smell of pit latrines was significantly associated with emptying frequency, household wealth status, residential type, and availability of piped water. In contrast, the occurrence of leakages was significantly associated with the lining condition of the pit, underscoring the importance of structural integrity for the facility’s performance. Wealthier households were more likely to better control pit latrine odor than low and middle-income households, suggesting better infrastructure or maintenance among wealthier households. Moreover, latrines used by households living in compounds with many other households were more likely to smell bad than those used in single houses, suggesting challenges in maintaining hygienic conditions of shared pit latrines.

#### 3.2.3 Meeting needs and expectations of users

Under the objective of Meeting Needs and Expectations of Users, cesspit users reported higher satisfaction compared to pit latrine users (76.4 against 27.1%) (Table 5). This finding could be explained by the fact that the physical condition of pit latrines exerts a more immediate influence on user experience compared to cesspits, which are generally located at a distance from the user interface, limiting the effect on user perception and experience. Additionally, sharing the facility with other households was more common among pit latrine users (62.6%) than cesspit users (7.2%), which could also explain the lower satisfaction among pit latrine users. Consequently, cesspits exhibited a slightly higher full compliance with Meeting Needs and Expectations of Users than pit latrines (11% against 4.9%).

**Figure 10:**
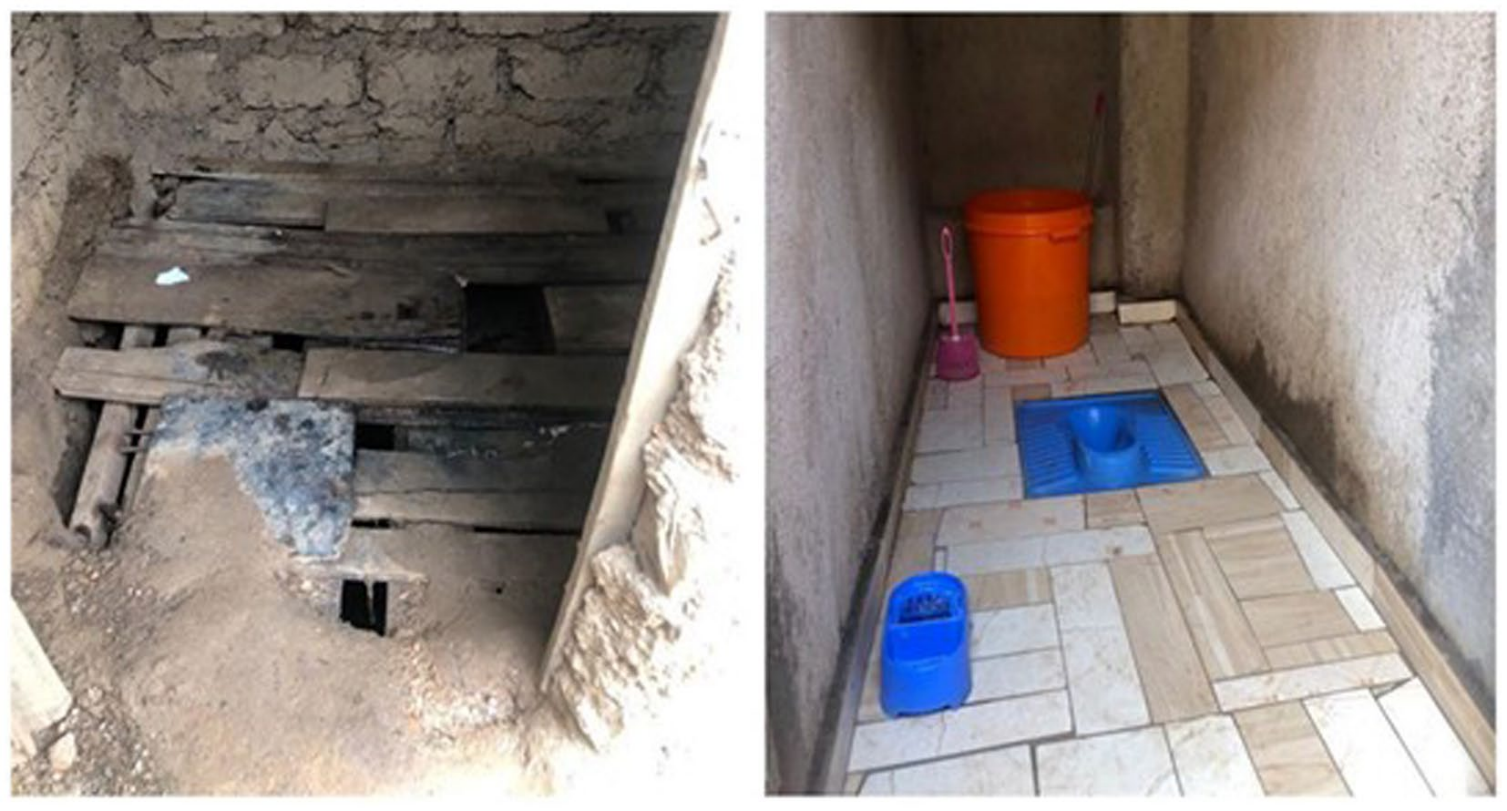
Pictures of pit latrines from the survey – (left photo) In very bad condition, (right photo) in good condition.

Figure 10 displays examples of inspected latrines during the survey; the left photo shows a pit latrine with a slab and pit in bad condition, where the slab is made of timber pieces with weak joints, spaces between them, and the pit provides minimal support to the slab. In contrast, the right photo shows a pit latrine with a slab and pit in good condition, offering limited risks of contact between users and feces.

For pit latrines, the results of GLMM in Table 6 suggested that very low-income and low-income households, and those living in compounds with many other households, were significantly more likely to be non-compliant with the standard objective Meeting Needs and Expectations of Users, compared to pit latrines in middle- and high-income households, and those in single-family houses. Pit latrines in households with very low income and those in rented houses were about five and three times, respectively, more likely to fall into non-compliance. Pit latrines in rented houses showed increased odds of not complying. In contrast, pit latrines in households without piped water were less likely to be non-compliant compared to those with piped water. This may be related to the non-reliance on piped water of pit latrines compared to cesspits, which generally receive blackwater from flushing. Random effect covariance suggested the potential divergence of full compliance across sectors.

Additionally, the results of GLMM showed that cesspits in very low-income households had lower odds of achieving full compliance with the standard objective Meeting Needs and Expectations of Users, compared to those in high-income households, whereas cesspits in middle-income households exhibited higher odds (Table 7). Cesspits in compounds with many households were associated with a reduced likelihood of compliance compared to those in single-family housing, as was the lack of piped water. Conversely, newer cesspits (<10 years) were significantly more likely to fully comply with this standard objective compared to older ones, suggesting a probable better intention to meet users’ needs and expectations in newly constructed cesspits. This aligns with the fact that those in the new settlement were also associated with full compliance. However, not owning a house was associated with decreased likelihood of full compliance, suggesting barriers related to house tenure. The random effects covariance suggested moderate clustering across sectors.

The analysis of the Rao-Scott Chi-Square associations (Table 9) showed that the extent to which a cesspit provided security to users was associated with pit conditions and the type of settlement. Cesspits in good condition and in old settlements provided users with more security than others. For pit latrines, the extent to which a latrine provided security to users was significantly associated with pit conditions, wealth, and house tenure. Households with middle to high income and those that owned the house were more likely to have pit latrines that provided security to users, suggesting that income and owning a house may be linked to the level of security offered by a latrine (Table 9). Lower-income households tended to share their pit latrine compared to middle and high-income households. Sharing facilities was also significantly associated with house tenure, where shared pit latrines were more likely to be found among tenants in compounds and low-income households than in wealthier households, owning or living in single-family houses. This finding suggests the likelihood of reduced access to sanitation among households living in compounds and those who are tenants.

**Table 9:** Rao-Scott chi-square analysis of associations between meeting needs and expectations of users characteristics and key technical and demographic characteristics.

Technical and demographic → Public health protection ↓			Sharing facility	Pit conditions	Depth of the pit	Emptying frequency	Lining status	Wealth	House tenure	Residential type	Settlement type	Presence of piped water	Age of the pit
Pit provides security to users	CP	Adjusted F	4.58	18.43*	1.16	1.17	0.00	1.42	0.00	4.53	14.01*	4.43	0.00
		Odds ratio	(0.78, 14.11)	(0.21, 0.76)	(0.33, 9.63)	(0.56, 3.61)	(0.38, 2.51)	(0.16, 2.15)	(0.62, 1.64)	(0.87, 2.89)	(0.26, 0.84)	(0.76, 19.23)	(0.19?4.83)
	PL	Adjusted F	3.27	44.55*	0.03	0.13	2.36	32.75*	7.10*	1.37	0.08	6.20	2.12
		Odds ratio	(0.85, 2.04)	(1.97, 5.41)	(0.47, 2.32)	(0.47, 1.77)	(0.23, 1.52)	(1.35, 2.42)	(0.96, 4.79)	(0.56, 4.01)	(0.53, 2.15)	(0.90, 5.35)	(0.79, 1.07)
Pit not shared with other households	CP	Adjusted F	–	0.55	0.27	4.76	1.01	1.16	0.00	17.71*	0.94	0.22	0.59
		Odds ratio	–	(0.69, 1.86)	(0.23, 2.66)	(0.10, 1.32)	(0.44, 5.27)	(0.26, 24.04)	(0.14, 7.31)	(1.75, 25.80)	(0.15, 2.51)	(0.02, 15.85)	(0.49, 3.40)
	PL	Adjusted F	–	0.66	8.18*	1.93	0.10	43.89*	12.18*	1.13	0.00	0.34	0.00
		Odds ratio	–	(0.45, 4.27)	(0.73, 0.99)	(0.70, 2.82)	(0.28, 2.69)	(1.58, 3.11)	(1.19, 5.08)	(0.65, 2.57)	(0.25, 3.79)	(0.42, 3.69)	(0.49, 2.14)
Users’ satisfaction with facility	CP	Adjusted F	2.41	0.67	20.95*	13.36*	3.77	0.51	3.95	0.98	52.20*	7.66*	1.14
		Odds ratio	(0.41, 8.26)	(0.68, 2.09)	(1.40, 6.19)	(1.18, 4.31)	(0.92, 1.56)	(0.42, 4.42)	(0.80, 3.90)	(0.66, 2.38)	(0.23, 0.53)	(1.02, 13.19)	(0.41, 1.46)
	PL	Adjusted F	8.02*	15.82*	0.78	9.11*	0.25	4.04	14.64*	3.47	1.77	12.59*	0.00
		Odds ratio	(1.01, 3.21)	(1.38, 11.37)	(0.36, 1.70)	(0.35, 0.97)	(0.54, 2.42)	(0.75, 5.38)	(1.22, 3.71)	(0.69, 6.30)	(0.78, 1.99)	(1.66, 11.96)	(0.38, 2.69)
Pit is not a danger to other houses	CP	Adjusted F	0.09	3.22	1.03	0.69	0.07	11.51*	0.94	0.09	4.80	0.07	12.75*
		Odds ratio	(0.03, 17.14)	(0.81, 2.72)	(0.22, 1.94)	(0.13, 36.33)	(0.22, 3.36)	(1.28, 5.89)	(0.62, 2.66)	(0.43, 2.84)	(0.19, 1.24)	(0.02, 23.46)	(0.07, 0.72)
	PL	Adjusted F	13.21*	251.32*	0.05	0.66	2.75	20.65*	4.24	1.49	0.19	47.78*	1.47
		Odds ratio	(1.15, 3.19)	(5.48, 12.05)	(0.39, 2.17)	(0.67, 2.03)	(0.17, 1.51)	(1.35, 3.65)	(0.78, 4.65)	(0.71, 2.38)	(0.58, 2.10)	(2.40, 7.83)	(0.38, 1.45)
Affordable	CP	Adjusted F	7.79*	2.32	0.84	0.27	2.96	2.34	0.39	8.81*	0.02	25.09*	34.93*
		Odds ratio	(0.23, 0.54)	(0.76, 2.46)	(0.53, 3.52)	(0.29, 2.30)	(0.75, 3.38)	(0.14, 1.71)	(0.42, 1.71)	(0.16, 0.93)	(0.44, 2.50)	(0.17, 0.59)	(1.74, 4.59)
	PL	Adjusted F	1.17	0.68	0.19	0.13	0.08	2.08	0.78	2.96	0.22	0.00	1.07
		Odds ratio	(0.37, 1.53)	(0.31, 1.87)	(0.44, 1.80)	(0.57, 1.53)	(0.47, 1.83)	(0.79, 2.07)	(0.40, 2.60)	(0.35, 1.27)	(0.39, 1.93)	(0.29, 3.17)	(0.52, 1.34)

Adjusted F: Rao-Scott Chi-square adjusted F statistic x2, Adjusted *F* values market in bold with * have *p* < 0.05.

The survey revealed that among households that shared their pit latrines, on average, a household shared a latrine with 3.4 other households (standard deviation = 2.4). The extent of sharing varied considerably, with the maximum number of households using a single latrine recorded at 16. A significant association was identified between sharing a pit latrine and user satisfaction, as well as the risk of the facility causing damage to neighboring households. Shared pit latrines were more likely to represent a danger to other neighboring houses and dissatisfy users than non-shared pit latrines.

The presence of piped water was significantly associated with user satisfaction, encompassing both cesspits and pit latrines (Table 9). Notably, pit latrines in households with piped water were regarded as safer, both for users and for neighboring households, and satisfying thereby reducing the perceived level of danger linked to their use and conditions. These findings underscore the role of water access in enhancing not only hygienic practices but also a broader sense of safety regarding sanitation facilities. For pit latrines, users were more dissatisfied with pits that had been emptied, and shallow pits were more likely to be shared. This suggests that emptying may be associated with users’ negative experience, while deep pits are more likely not shared, targeting long-term use without filling.

For cesspit users, the depth of the pit was associated with user satisfaction, where households with deeper cesspits were more satisfied than those with shallow cesspits – possibly because deeper pits may take longer to fill, reducing frequent reliance on emptying services and related financial cost. Emptying frequency was also associated with sharing the cesspit, indicating that not sharing the cesspits may decrease the likelihood of emptying them frequently. However, cesspits constructed within the last 10 years demonstrated a significant association with being perceived as likely affordable and dangerous to surrounding houses. This pattern suggests that recent construction practices may have involved less controlled or regulated development, thereby increasing risks to neighboring households. This finding contrasts with the likelihood of newer cesspits demonstrated in complying with the standard objective, Meeting Needs and Expectations of Users. The association with individual characteristics of the standard objectives suggests underlying factors, for example, that new construction practices may focus more on affordability and satisfaction.

#### 3.2.4 Sustainability

**Figure 11:**
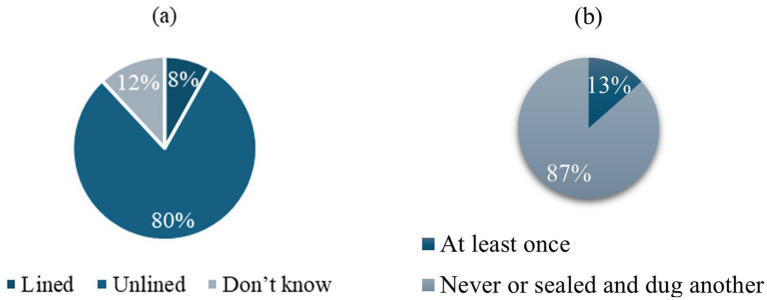
**(a)** Pit latrine lining status (%), **(b)** emptying frequencies (%).

**Figure 12:**
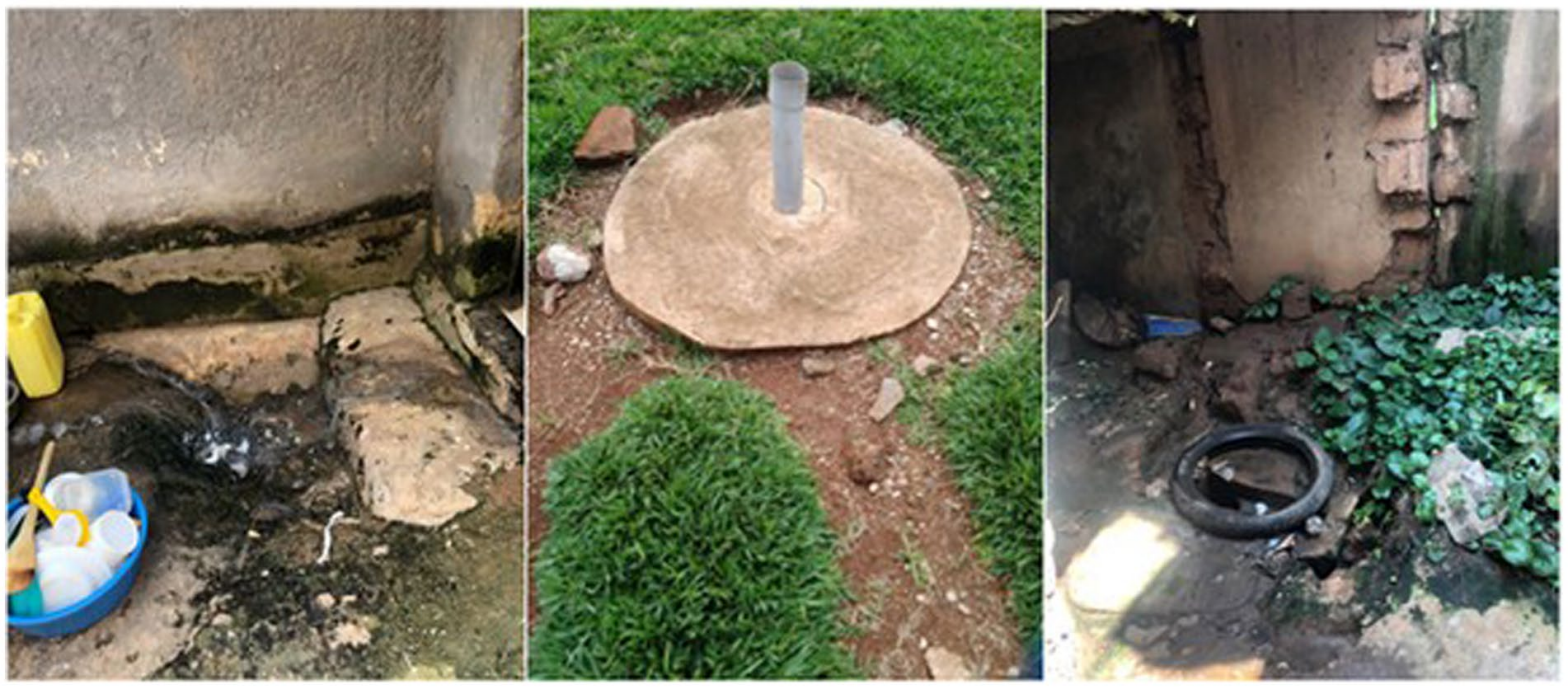
Pictures of cesspits from the survey – (Left photo) Cesspit used to capture excreta, stormwater, and other household wastewater; (Middle photo) cesspit in good condition, well covered, hygienic, located far apart in the garden; (Right photo) cesspit in very bad condition, unhygienic, partly covered.

Under this standard objective, results show that full compliance of both cesspits and pit latrines was equal, scoring 0.4% (Table 5) – suggesting that very few facilities may be sustainable. For both types of facilities, the performance was notably low for most characteristics. For example, the proportion of pits without lining was 91.1% for pit latrines and 78.5% for cesspits; while those that were never emptied was 86.5% for pit latrines and 86.9% for cesspits (Table 5, Figure 11). Superstructure and pits in bad conditions were mainly characterized by physical damage, old structural components, and a visible lack of maintenance (Figure 12). For example, some cesspits were covered with half concrete and other materials, all seemingly old, with grass growing around, presenting potential risks of causing accidents. Some other cesspits serve as multipurpose pits and capture different types of wastewater from the household.

Furthermore, 0.4% compliance with this objective suggests that this may not be dependent on the investigated demographic characteristics, but rather may be rooted in the other underlying factors, such as sanitation governance system-wide gaps, especially in planning, technology promotion, regulation enforcement, social norms, behavior factors, and standards implementation (30). Moreover, the findings under this objective suggest that there may have been a widely implemented one-size-fits-all solution without further adaptation to sustainability requirements.

The results of the Rao-Scott Chi-Square analysis are presented in Table 10. In pit latrines, pit conditions were significantly associated with the presence of reinforced concrete slabs, superstructures in good condition, proper rainwater drainage, and users’ knowledge of sanitation regulations. Pits in good condition were more likely to have reinforced concrete slabs, latrines with superstructures in good condition, and not to drain rainwater into the pit. Moreover, individuals who reported being aware of sanitation regulations tended to have pit latrines with both pits and slabs in good condition, suggesting a potential positive association with knowledge of regulations of sanitation facilities maintenance.

**Table 10:** Rao-Scott chi-Square analysis of associations between Sustainability characteristics and key technical and demographic characteristics.

Technical and demographic → Public health protection ↓			Sharing facility	Pit conditions	Depth of the pit	Emptying frequency	Lining status	Wealth	House tenure	Residential type	Settlement type	Presence of piped water	Age of the pit
Reinforced Concrete Slab	CP	Adjusted F	5.23	0.74	0.15	12.84*	0.43	1.66	5.74	1.56	2.06	0.36	0.13
		Odds ratio	(0.90, 5.40)	(0.11, 3.18)	(0.48, 1.73)	(1.07, 1.82)	(0.27, 7.86)	(0.06, 3.11)	(0.19, 1.18)	(0.76, 2.00)	(0.70, 2.97)	(0.01, 16.40)	(0.46, 1.79)
	PL	Adjusted F	0.05	155.06*	0.00	0.38	1.46	36.92*	0.54	1.24	0.44	29.42*	0.18
		Odds ratio	(0.45, 2.55)	(2.69, 4.80)	(0.28, 3.58)	(0.39, 4.32)	(0.33, 1.53)	(1.74, 4.71)	(0.44, 4.00)	(0.59, 3.32)	(0.48, 3.16)	(2.32, 14.94)	(0.73, 1.53)
Lined pit	CP	Adjusted F	1.01	0.04	0.05	0.35	–	11.30*	0.54	0.01	0.08	0.05	18.06*
		Odds ratio	(0.44, 5.27)	(0.48, 1.87)	(0.31, 2.67)	(0.40, 1.78)	–	(0.48, 0.92)	(0.62, 2.22)	(0.47, 1.95)	(0.53, 2.17)	(0.21, 6.20)	(1.23, 2.67)
	PL	Adjusted F	0.10	1.79	0.00	0.41	–	1.30	0.48	2.32	0.40	0.14	0.63
		Odds ratio	(0.28, 2.69)	(0.18, 1.79)	(0.26, 3.54)	(0.36, 5.20)	–	(0.57, 3.73)	(0.32, 1.98)	(0.82, 1.85)	(0.06, 5.65)	(0.21, 7.65)	(0.40, 5.19)
Superstructure in good condition	CP	Adjusted F	–	–	–	–	–	–	–	–	–	–	–
		Odds ratio	–	–	–	–	–	–	–	–	–	–	–
	PL	Adjusted F	5.14	599.47*	0.14	1.98	0.94	11.56*	5.91	2.08	0.02	43.46*	0.71
		Odds ratio	(0.88, 3.00)	(18.75, 45.97)	(0.29, 2.55)	(0.45, 1.29)	(0.21, 2.11)	(1.20, 8.51)	(0.88, 5.44)	(0.76, 2.34)	(0.43, 2.09)	(2.51, 10.44)	(0.63, 2.33)
Slab and pit in good condition	CP	Adjusted F	0.55	–	0.19	0.57	0.43	152.18*	0.44	0.02	0.01	0.59	0.00
		Odds ratio	(0.69, 1.86)	–	(0.34, 2.10)	(0.31, 2.66)	(0.48, 1.87)	(2.02, 3.04)	(0.61, 2.22)	(0.60, 1.75)	(0.54, 1.75)	(0.5, 5.52)	(0.73, 1.33)
	PL	Adjusted F	0.66	–	0.10	2.44	1.79	5.69	5.59	0.04	0.01	60.90*	4.78
		Odds ratio	(0.45, 4.27)	–	(0.54, 2.16)	(0.55, 1.17)	(0.18, 1.79)	(0.91, 3.11)	(0.87, 4.79)	(0.53, 2.07)	(0.49, 2.17)	(3.11, 12.16)	(0.89, 2.56)
Emptying frequency	CP	Adjusted F	4.76	0.05	70.07*	–	0.35	0.16	1.09	2.16	3.02	4.92	13.89*
		Odds ratio	(0.10, 1.32)	(0.31, 2.66)	(2.41, 6.06)	–	(0.40, 1.78)	(0.17, 3.69)	(0.54, 1.32)	(0.72, 2.85)	(0.14, 1.56)	(0.51, 35.14)	(0.05, 0.76)
	PL	Adjusted F	1.93	2.44	15.87*	–	0.41	1.17	4.91	5.14	0.03	0.04	64.74*
		Odds ratio	(0.70, 2.82)	(0.55, 1.17)	(1.45, 8.37)	–	(0.36, 5.20)	(0.78, 1.74)	(0.91, 2.29)	(0.85, 4.52)	(0.39, 2.26)	(0.45, 1.95)	(0.04, 0.25)
Rainwater does not flow into pit	CP	Adjusted F	2.88	1.56	29.02*	3.18	1.84	0.00	0.00	0.08	2.67	2.23	0.15
		Odds ratio	(0.04, 2.66)	(0.36, 1.47)	(0.18, 0.55)	(0.30, 1.29)	(0.19, 2.54)	(0.15, 5.75)	(0.35, 2.66)	(0.50, 2.33)	(0.50, 1.19)	(0.65, 4.62)	(0.44, 1.83)
	PL	Adjusted F	0.18	450.74*	0.24	0.01	0.03	2.26	0.00	0.01	0.13	38.88*	1.14
		Odds ratio	(0.46, 2.86)	(3.34, 4.87)	(0.27, 2.43)	(0.30, 2.97)	(0.22, 3.75)	(0.60, 5.35)	(0.33, 3.06)	(0.32, 3.29)	(0.38, 3.46)	(1.93, 5.38)	(0.92, 1.19)
Only excreta (for pit latrines) and black water (for cesspits) are discharged into pit	CP	Adjusted F	3.40	0.00	55.03*	6.26	2.63	24.79*	2.37	15.31*	10.57*	0.36	1.93
		Odds ratio	(0.65, 10.24)	(0.36, 2.77)	(0.28, 0.55)	(0.43, 1.04)	(0.27, 1.38)	(1.91, 8.90)	(0.67, 3.97)	(1.26, 3.91)	(0.39, 0.93)	(0.27, 7.60)	(0.35, 1.40)
	PL	Adjusted F	0.00	0.00	2.94	2.36	0.03	4.55	0.00	1.15	(3.64)	0.24	0.53
		Odds ratio	(0.48, 2.15)	(0.40, 2.35)	(0.71, 3.87)	(0.58, 5.87)	(0.32, 3.58)	(0.30, 1.17)	(0.37, 2.62)	(0.43, 1.44)	(0.83, 2.85)	(0.24, 2.69)	(0.34, 1.86)
Pit cover has opening for emptying services	CP	Adjusted F	5.27	0.98	2.58	1.05	0.00	0.00	21.58*	37.95*	0.25	1.29	0.00
		Odds ratio	(0.76, 7.61)	(0.41, 1.52)	(0.77, 2.69)	(0.65, 2.53)	(0.31, 2.93)	(0.16, 5.99)	(1.26, 2.64)	(1.51, 3.11)	(0.57, 1.47)	(0.50, 4.88)	(0.68, 1.42)
	PL	Adjusted F	0.05	3.77	12.27*	1.96	1.93	1.19	0.29	4.94	0.57	0.85	4.10
		Odds ratio	(0.31, 2.64)	(0.82, 2.87)	(1.36, 9.49)	(0.45, 11.59)	(0.50, 9.30)	(0.38, 1.51)	(0.29, 2.28)	(0.88, 2.99)	(0.15, 2.88)	(0.67, 2.16)	(0.11, 1.47)
Residence is accessible by road at least 3 m wide	CP	Adjusted F	2.00	0.09	0.07	0.63	0.41	4.31	7.95*	1.42	12.15*	26.33*	4.59
		Odds ratio	(0.48, 11.60)	(0.64, 1.72)	(0.19, 7.14)	(0.36, 1.74)	(0.16, 17.71)	(0.83, 4.35)	(1.02, 7.37)	(0.47, 6.74)	(0.21, 0.86)	(3.32, 50.86)	(0.92, 1.74)
	PL	Adjusted F	0.31	0.21	2.61	0.07	0.76	0.96	0.12	16.53*	0.35	1.61	0.25
		Odds ratio	(0.71, 1.65)	(0.62, 1.39)	(0.13, 1.68)	(0.53, 1.67)	(0.32, 8.23)	(0.81, 1.53)	(0.85, 1.22)	(1.18, 2.60)	(0.17, 3.06)	(0.62, 3.66)	(0.55, 2.33)
Pit can be easily accessed for emptying services	CP	Adjusted F	0.03	1.56	5.28	12.13*	6.65	2.24	14.25*	1.68	70.66*	8.71*	16.60*
		Odds ratio	(0.24, 4.91)	(0.31, 1.55)	(0.62, 27.30)	(1.18, 5.07)	(0.55, 1.02)	(0.52, 8.40)	(1.20, 3.54)	(0.72, 2.45)	(0.16, 0.40)	(1.00, 10.25)	(0.21, 0.75)
	PL	Adjusted F	0.52	1.08	3.28	2.31	0.88	0.12	0.23	6.00	6.43	26.87*	1.08
		Odds ratio	(0.64, 2.13)	(0.63, 2.73)	(0.61, 9.70)	(0.71, 3.09)	(0.02, 6.33)	(0.95, 0.67)	(0.56, 2.23)	(0.94, 2.64)	(0.09, 1.13)	(1.12, 1.46)	(0.32, 1.67)
Knowledge of regulations about toilet construction	CP	Adjusted F	0.788	2.67	0.00	0.59	0.26	0.54	51.96*	0.24	5.39	5.00	2.09
		Odds ratio	(0.48, 3.95)	(0.52, 1.17)	(0.31, 3.37)	(0.43, 1.59)	(0.62, 2.00)	(0.33, 6.51)	(1.72, 3.56)	(0.45, 3.05)	(0.34, 1.10)	(0.75, 7.32)	(0.75, 2.45)
	PL	Adjusted F	0.74	35.98*	0.03	6.12	0.63	49.94*	2.51	2.16	1.18	5.47	0.49
		Odds ratio	(0.47, 4.12)	(1.78, 6.12)	(0.17, 7.49)	(0.96, 2.05)	(0.36, 5.20)	(2.60, 12.60)	(0.80, 2.23)	(0.11, 2.04)	(0.64, 1.20)	(0.82, 5.03)	(0.33, 1.90)

Adjusted F: Rao-Scott Chi-square adjusted F statistic x2, Adjusted *F* values market in bold with * have *p* < 0.05.

Depth of the pit was associated with emptying frequency, not draining rainwater into the pit, and using the cesspit only for blackwater management. Households that had never emptied, sealed, or dug another pit after the old one was filled had deeper pits (more than 10 m) compared to others. Similarly, deeper cesspits were more likely to drain rainwater and other household effluents than shallower ones. This suggests that households may adopt deeper pits to avoid emptying them sooner and use them to dispose of different households’ wastewater, including rainwater.

Wealth was significantly associated with the type of slab, condition of the latrine superstructure, and knowledge of sanitation regulations. Middle to wealthier households were more likely to have pit latrines with a reinforced concrete slab and superstructure in good condition, and to be aware of sanitation regulations compared to households with very low and low-income. Moreover, middle to wealthier households were more likely to have lined cesspits in good condition, and only use them to manage blackwater. This finding suggests that high income may play a critical role in better access to information related to sanitation and facilities in good condition, therefore underscoring inequities in regulations awareness and facility maintenance capability across socioeconomic strata.

For cesspits, the presence of an opening for emptying was significantly associated with house tenure and residential type, with owners and single-family homes more likely to have cesspits with openings designated for emptying than tenants and compounds with many households. This could indicate that rental houses are more likely to have cesspits not designed for future emptying services. Conversely, settlement type and the presence of piped water were significantly associated with whether a household was accessible by a road at least 3 m wide and whether a cover for emptying services was present. Households in new settlements and those with piped water were more likely to be accessible by a road at least 3 m wide and to have slabs with openings for emptying services. This suggests that households in older settlements may face barriers related to access to piped water and emptying services, highlighting the importance of access to water supply and road infrastructure for improved sanitation.

Similarly, the presence of piped water was significantly associated with pit latrines with reinforced concrete slabs, superstructure in good conditions, slab and pit in good conditions, not draining rainwater into the pit, and easy access to the pit for emptying services, further underscoring the critical role of access to water on improved sanitation at households.

Additionally, lining status showed a significant association with pit age and emptying frequency among cesspit users, suggesting that less aged pits are more likely to be lined and emptied more frequently. This may also explain progress in planning future emptying services, especially in new settlements where pits are more likely to be lined than in old settlements. The contradiction to this finding observed under the standard objective Meeting Needs and Expectations of Users may reflect a divergence in emerging sanitation construction practices.

#### 3.2.5 Protection of the environment

The overall full compliance with this standard objective was very low – 3.4% for pit latrines and 2.0% for cesspits (Table 5). This was mainly associated with high proportions of pits with depth over 10 m (82.8% for pit latrines and 88.9% for cesspits), and limited sanitary inspections by authorities (72.0% for pit latrines and 82.7% for cesspits) (Table 5).

In Table 6, the GLMM results showed that pit latrines in very-low, low, and middle-income households were significantly more likely (nine, eleven, and ten times, respectively) to be non-compliant with the standard objective Protection of Environment compared to those in high-income households. In contrast, pit latrines in new settlements, rented housing, and those in households without piped water were more likely to be fully compliant with this standard objective, suggesting that these factors may be linked to the adoption of characteristics that promote protection of the environment. Random effects exhibited a strong positive association, suggesting that sectors may be associated with compliance with this standard objective.

On the other hand, cesspits in very low and low-income households had lower odds of non-compliance compared to those in high-income households, whereas those in middle-income households had higher odds (Table 7). Similarly, cesspits in newer settlements were significantly associated with higher odds of non-compliance, while those in rented housing were associated with lower odds of non-compliance. This finding suggests that very low to low income and housing for rent may facilitate the adoption of cesspits with characteristics that promote the protection of the environment.

In Table 11, Rao-Scott Chi-Square results show that pit latrines in households with high and low income, living in compounds with other households, were more likely to be inspected by government agents than those in middle-income households, suggesting limited inclusivity in the sanitation inspection framework in place, where households living in shared compounds were more inspected than others. In contrast, households in single houses were subjected to fewer inspections by government agencies. Conversely, users of cesspits within compound settings tended to experience more inspections.

**Table 11:** Rao-Scott chi-Square analysis of associations between protection of environment characteristics and key technical and demographic characteristics.

Technical and demographic → Public health protection ↓			Sharing facility	Pit conditions	Depth of the pit	Emptying frequency	Lining status	Wealth	House tenure	Residential type	Settlement type	Presence of piped water	Age of the pit
Depth of pit	CP	Adjusted F	0.27	0.23	–	70.07*	0.05	8.44*	1.48	0.33	0.00	0.01	6.35
		Odds ratio	(0.23, 2.66)	(0.34, 2.10)	–	(2.41, 6.06)	(0.31, 2.67)	(0.26, 0.93)	(0.58, 3.83)	(0.42, 3.64)	(0.40, 2.55)	(0.93, 0.13)	(0.05, 1.41)
	PL	Adjusted F	8.18*	0.10	–	15.87*	0.00	3.10	0.06	3.07	0.45	0.39	35.39*
		Odds ratio	(0.73, 0.99)	(0.54, 2.16)	–	(1.45, 8.37)	(0.26, 3.54)	(0.49, 1.17)	(0.402, 2.12)	(0.65, 6.44)	(0.32, 1.98)	(0.62, 2.11)	(0.17, 0.55)
Ground- or soil-water not reached while digging pit	CP	Adjusted F	0.59	0.22	11.94*	1.47	0.24	0.10	0.00	0.94	6.27	0.09	7.66*
		Odds ratio	(0.48, 3.64)	(0.31, 2.28)	(1.14, 8.14)	(0.62, 3.36)	(0.53, 1.54)	(0.23, 6.14)	(0.46, 2.15)	(0.77, 1.72)	(0.27, 1.07)	(0.18, 8.8.14)	(0.34, 0.99)
	PL	Adjusted F	4.40	0.13	9.36*	0.06	14.97*	0.66	8.01*	0.46	0.53	1.81	9.88*
		Odds ratio	(0.90, 2.08)	(0.67, 1.66)	(1.06, 7.02)	(0.29, 2.81)	(0.24, 0.81)	(0.57, 2.73)	(0.65, 0.99)	(0.75, 1.59)	(0.55, 2.75)	(0.76, 2.17)	(0.34, 0.93)
Authorities inspect pit	CP	Adjusted F	0.39	1.36	0.02	0.00	0.49	11.66*	0.45	17.45*	0.96	28.44*	4.48
		Odds ratio	(0.33, 1.96)	(0.38, 1.46)	(0.55, 1.91)	(0.54, 1.80)	(0.10, 4.01)	(0.15, 0.78)	(0.50, 1.50)	(0.38, 0.82)	(0.62, 2.65)	(0.43, 0.75)	(0.26, 1.22)
	PL	Adjusted F	1.61	3.56	0.00	2.81	0.11	0.19	2.59	15.84*	0.38	1.04	0.46
		Odds ratio	(0.61, 3.69)	(0.77, 3.56)	(0.41, 2.45)	(0.75, 3.18)	(0.17, 3.88)	(0.49, 2.67)	(0.61, 6.21)	(1.16, 2.34)	(0.49, 2.99)	(0.47, 4.88)	(0.69, 1.84)

Adjusted F: Rao-Scott chi-square adjusted F statistic x2, Adjusted *F* values market in bold with * have *p* < 0.05.

Cesspits of <10 years were more likely to be deeper and less inspected by authorities, emphasizing gaps in inspections during the construction of sanitation facilities, as suggested earlier. Deeper pit latrines (>10 m) were more likely to be older (more than 10 years), used by households that owned the house, and had a higher likelihood of having reached groundwater or moist soil when they were dug. For both pit latrines and cesspits, older pits had more chances of having reached groundwater during digging. This may reflect the potential groundwater contamination among old pit latrines and cesspits.

A total of 93.2% pit latrine users and 80.2% of cesspit users reported having additional pits within their compounds specifically for the disposal of stormwater and other effluents generated at the household level. This finding suggests that pits may serve as primary facilities for managing wastewater generated at the household level. It may also suggest potential soil moisture saturation and increased emanating risks.

### 3.3 Recommendations by households

**Figure 13:**
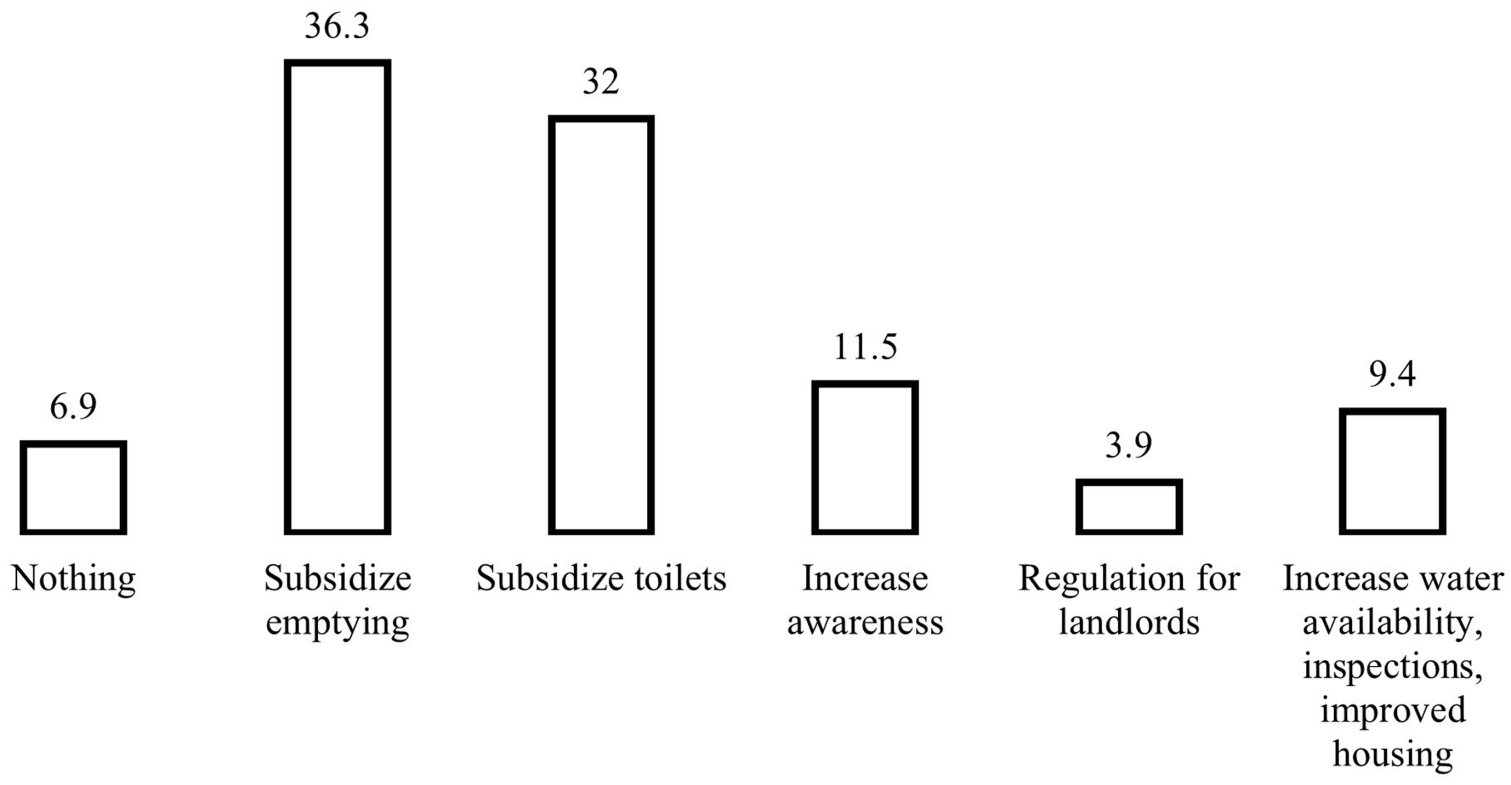
Recommendations by pit latrine users for enhanced sanitation (%).

**Figure 14:**
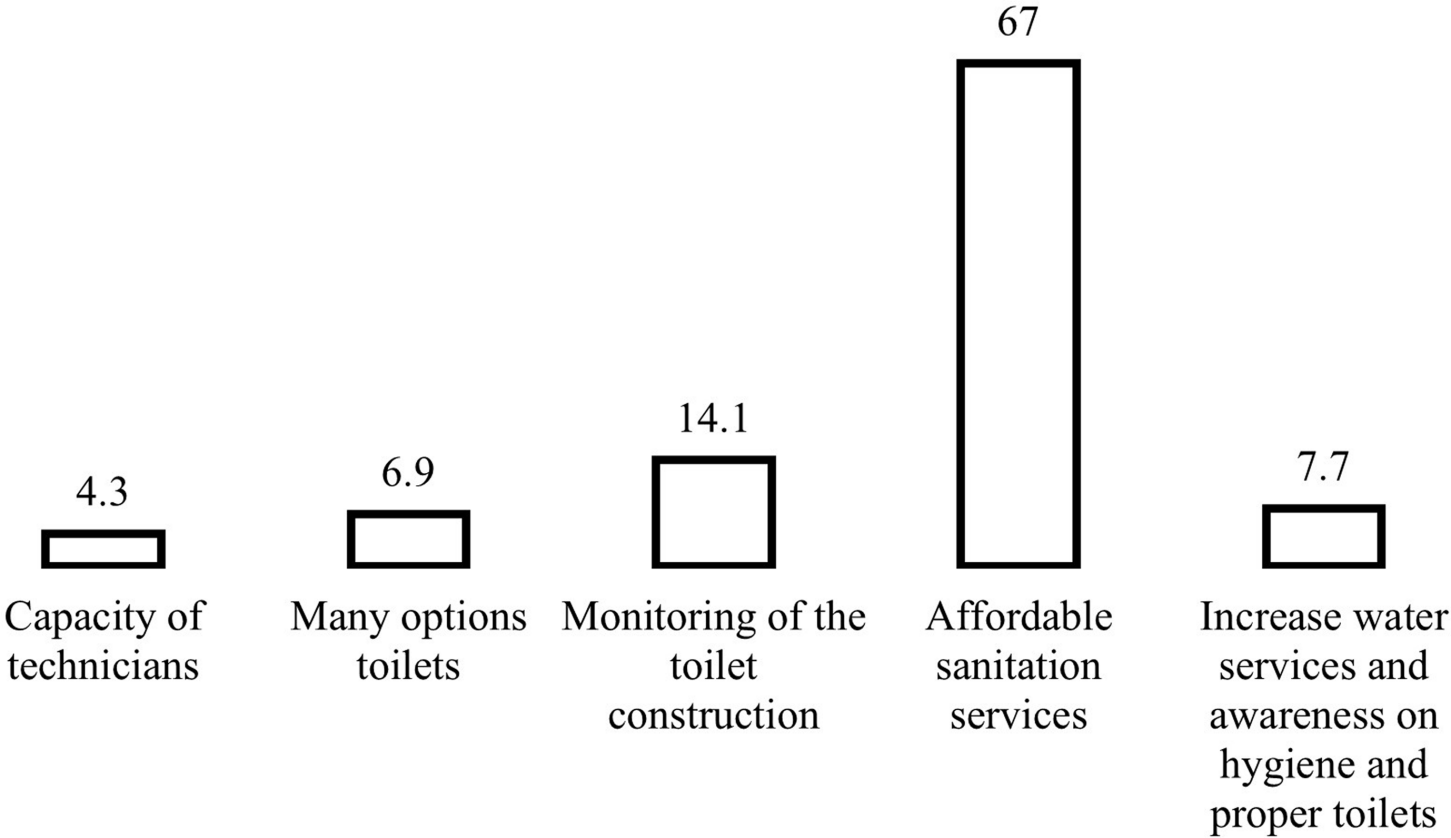
Recommendations by cesspit users for enhanced sanitation (%).

During the survey, participants provided recommendations on the measures that they think the government should take to enhance access to safe sanitation services. Recommendations from pit latrine users predominantly emphasized the need for government intervention through subsidies for toilet construction and emptying services (Figure 13). On the other hand, cesspit users mainly recommended establishing affordable emptying services and an effective monitoring framework of toilet construction (Figure 14).

Other recommendations by respondents included increasing public awareness, strengthening the capacity of technicians, providing alternative technologies, and enhancing water supply services, which all align with current water and sanitation policy actions (31). Among pit latrine users, 3.9% recommended regulating the responsibilities of landlords regarding sanitation services. This recommendation stems from challenges in delineating accountability for the operation and maintenance of shared latrines, including emptying, cleaning, and structural upkeep; issues that were closely linked to facility sharing, house tenure, and the physical condition of the pit latrine.

## 4 Discussion and recommendations

### 4.1 Summary of compliance status and key gaps

This study found that domestic pit latrines and cesspits had high non-compliance levels with RS ISO24521. Full Compliance with the standard objectives Protection of Public Health, Meeting Needs and Expectations of Users, Sustainability, and Protection of Environment was 0.5, 4.9, 0.3 and 3.4%, respectively, for pit latrines; and 79.4, 11, 0.4 and 2% for cesspits. While Moderate and Low Compliance were relatively evenly distributed across the selected objectives among pit latrines, the distribution was uneven among cesspits. However, reliance on self-reported and observed information, subject to recall and reporting bias, may have led to misclassification in these compliance estimates, and they should be interpreted with this in mind.

Identified key gaps in surveyed households pit latrines and cesspits include: limited sanitary inspections, widespread use of unsustainable pit latrines and cesspits offering limited protection of public health and environment, limited financing mechanisms for households’ sanitation facilities and services, high cost of sanitation services, low public awareness of proper sanitation and hygiene practices, limited options of appropriate sanitation technologies, insufficient technical capacity and unsustainable water supply services potentially undermining the effectiveness of sanitation practices.

The observed patterns in the choice of sanitation facility type could indicate that factors such as affordability, popularity, peer imitation, and the reliability of the water supply may shape decisions regarding the adoption of pit latrines. Affordability of sanitation services, sanitation market dynamics, and social norms were proven by other researchers as important factors influencing sanitation behaviors, particularly in middle and low-income countries (4, 32).

Across this study, both types of OSS in lower-income households consistently exhibited reduced likelihood of achieving full compliance, especially with the standard objective Protection of Public Health (cesspits) and Meeting Needs and Expectations of Users (both), aligning with global evidence showing that financial constraints limit households’ ability to access sanitation (2). Similarly, in multi-country analyses, studies have reported that wealth is one of the factors influencing sanitation adoption and use (33–35). This could indicate income-based disparity in accessing improved sanitation, which tends to be a global and regional trend, highlighting the need for inclusive and income-sensitive interventions (2, 36, 37). These findings accord with (89), who emphasized that barriers to water, sanitation, and hygiene disproportionately affect the urban poor, reinforcing sanitation poverty as a system-wide challenge.

Housing type and tenure also emerged as important factors influencing full compliance. Cesspits and pit latrines in compound settings were generally less likely to achieve full compliance with standard objectives, Protection of Public Health (cesspits) and Meeting Needs and Expectations of Users (both OSS). This may reflect challenges related to shared responsibilities for sanitation infrastructure and unclear accountability for maintenance. Previous studies have documented that shared sanitation facilities are often associated with lower maintenance standards compared to private facilities (38). Similarly, (88) further highlighted how tenure insecurity in informal settlements complicates accountability for sanitation services, echoing this study’s finding that suggested how landlord responsibilities may often be neglected. This evidence may reflect that sanitation poverty in urban areas is not only a matter of infrastructure deficits but also of institutional and regulatory gaps.

The role of settlement type and cesspit age highlighted structural associations. Cesspits in new settlements and newer cesspits were likely to fully comply with standard objectives, Protection of Public Health (cesspits), and Meeting Needs and Expectations of Users. This could indicate better planning and integration of practices that promote full compliance. These findings suggest that upstream planning and regulatory enforcement can be critical for achieving better sanitation outcomes (30).

Access to piped water also appeared to be an important enabling factor. For standard objectives, Protection of Public Health (cesspits), and Meeting Needs and Expectations of Users, in both cesspits and pit latrines, it was significantly associated with non-compliance, in alignment with the established interdependence of water supply and sanitation (35, 39).

Random effects covariances varied highly across outcomes, suggesting that full compliance is partly associated with clustering at the sector level. This may reflect contextual factors such as sanitation governance, service provision, and social norms. This aligns with other studies that have documented that social and cultural practices require motivation and stakeholders’ support in creating sustainable sanitation behaviors at the community level (40, 41).

Key characteristics that underlie these gaps and may be linked to low compliance for both pit latrines and cesspits include unlined and very deep pits, low emptying frequencies, a lack of opening for emptying services, limited knowledge of regulations, and a lack of sanitary inspections. For pit latrines, in addition to those characteristics, uncleanness, bad smell, user dissatisfaction, sharing toilets, and difficult access to the pit for emptying were highly linked to low compliance of pit latrines.

These results could indicate a persistent deficiency in the design and operation of households’ pit latrines and cesspits. Other sources have proven that quality containment provides safe storage of fecal sludge, preventing contact with humans and the environment at the point of generation, and thus becomes central to adequate fecal sludge management (3, 78). Therefore, FS containment design is an important technical dimension that plays a key role in safe FS containment and desludging (2, 42).

Limited sanitary inspections and a lack of knowledge of sanitation regulations among respondents, as demonstrated by this study, may reflect how compliance could be linked to a lack of routine performance audits and the absence of regulatory enforcement. This aligns with previous studies that highlighted how gaps in community awareness and institutional capacity could contribute to the prevalence of substandard OSSs and limited demand for professional emptying practices (25, 43). These findings further align with studies from Kenya and Uganda that pointed to poorly regulated pit latrine construction as a driver of poorly performing facilities and environmental contamination (44, 45). They also corroborate guidelines from WHO that emphasize how sanitary inspections help ensure community involvement, compliance, risk identification and reduction, public health protection, and data collection for better planning (25). For example, this study suggests that households living in compounds are more inspected, yet they are also the ones with deeper pits, suggesting the potential ineffectiveness of conducted inspections.

Among surveyed facilities, this study found that sharing pit latrines, their bad smell, and their unhygienic status decreased satisfaction among users and the safety of the facility. This corroborates previous research indicating that sharing a pit latrine among multiple households contributes to increased dissatisfaction, poor maintenance, and a higher risk of disease transmission resulting from unhygienic conditions (46, 47). Similarly, other studies have demonstrated that smell, cleanliness, safety, sharing, and filling up quickly are the main drivers of dissatisfaction with pit latrines (48–50). While the poor conditions of the pit and slab may lead to leaking and sliding, the physical state of the superstructure can also heighten feelings of insecurity and reduce privacy for users (Figure 5). These findings further corroborate Nakagiri et al. (45), who reported that pit latrines in urban areas frequently underperform due to rapid filling, odor, insect nuisance, and poor maintenance, with compliance gaps in construction and oversight (45). This study has revealed that a lack of lining may increase the likelihood of leaking to the surrounding environment, therefore increasing risks of contaminating people and the environment with fecal pathogens (85). Limiting the sharing of sanitation facilities between households and improving the quality of the structure could enhance users’ satisfaction. These outcomes mirror regional evidence highlighting that pit latrines often underperform due to inadequate oversight and weak enforcement of construction standards and routine maintenance standards (51).

Moreover, this study suggests that the unsustainability of pit latrines and cesspits may be linked to issues related to key operational and design features, such as depth of pit, lining, superstructure components, and emptying frequencies. This accords with other studies conducted on the sustainability of decentralized wastewater treatment plants in Kigali. Their findings revealed that the sustainability and performance of decentralized wastewater treatment plants were hindered by issues related to operational and management factors (7, 52–54), suggesting a potential challenge in the operation and management of sanitation facilities. However, the root cause may be further linked to limited operationalization and enforcement of regulations (7, 13, 45). Evidence from other sources shows that lining pits may contribute to improved emptying services by increasing the demand for emptying due to limited infiltration, reducing environmental contamination, and providing structural integrity during emptying, among other benefits (21, 42, 78).

### 4.2 Health risks

Low full compliance with selected objectives of RS ISO 24521 may reflect the challenges encountered by households in accessing safely managed sanitation, potentially due to the limited availability of choices of technologies and services. For example, this study has found that most investigated latrines did not comply with the Protection of Public Health and Meeting Needs and Expectations of Users. This may indicate that they may likely not be appropriate for children, older adults people, and people with disabilities, potentially increasing their exposure to hazards such as accidents or fecal contamination, and a lack of privacy, particularly for women and girls (83, 80, 27, 28). Williams and Overbo (55) have demonstrated that households using poor-performing systems have increased exposure to pathogens associated with diarrheal diseases. In this study, surveyed pit latrines were found to be particularly unhygienic (89.2%) and generally without a handwashing facility nearby (90.8%), which suggests that there might be limited toilet hygiene and handwashing practices after usage, which in turn presents high risks of disease transmission (56–58, 84). The absence of these two hygiene practices is closely linked to diarrheal diseases, which are the third leading cause of death and malnutrition among children under 5 years globally (59). These findings accord with a study conducted in Rwanda in 2021 on excreta-related diseases, which suggested that Shigella and Enterotoxigenic *E. coli* are the main causes of childhood diarrheal disease, and highlighted that rotavirus remains a significant cause of diarrhea in Rwanda despite progress made in vaccination and access to water and sanitation (60). Moreover, this study showed that 50.3% of surveyed pit latrines had flies around the pit, potentially raising the risk of fecal oral disease transmission for both users and the community, as infected feces can be transported out of households by flies (58).

Additionally, this study found that most investigated pit latrines and cesspits were deep and unlined, suggesting potential infiltration of wastewater in the soil. This may pose health risks, particularly to the about 15.7% (approximately 274,000) people in Kigali who rely on wells, protected and unprotected springs, and surface water as their primary source of water (8). This suggestion aligns with a study conducted in 2022 in Kigali, which suggested that pit latrines are potential pollutants of protected springs (61). This could indicate that unlined and leaking pits may be associated with increased soil moisture, which, combined with favorable temperatures, could create a bacterial hotspot, potentially raising the risk of fecal-oral contamination through various routes (55). Furthermore, saturated soil may facilitate soil sliding into an unlined pit, mixing with sludge, and making subsequent de-sludging operations more unhygienic and difficult (42).

Respondents also indicated that an unsustainable water supply may affect their sanitation practices. This finding aligns with a previous study conducted in Kigali, which indicated that water supply shortage in the city was a concern, whereby water availability, sanitation, and hygiene were found strongly associated with households’ wellbeing (62). A recent study in Ghana similarly found that water scarcity affects sanitation and hygiene practices (63). In fact, reliable and sustained water supply services support sanitation infrastructure and are key enablers of proper sanitation and hygiene practices, contributing to a healthier and productive community (28, 64, 65).

### 4.3 Opportunities for enhanced sanitation services

The findings of this study suggest that relying only on pit latrines and cesspits in their current status may not provide the desired resilience, sustainability, and health outcomes. Moreover, the observed adoption of lining and provision of opening for emptying services among less-aged pits demonstrates an emerging will among users to include emptying services in design. This may reflect a need and interest for diversified sanitation services, which are considered an effective enabler to improve access to sanitation services and address issues related to inequalities, rapid urbanization, and climate change (65–67). Previous studies have demonstrated that communities are willing to pay for sanitation services; however, facilities are difficult to service, and the cost of services remains high for most households (Gahima et al., 2021) (9, 30, 68). To support inclusive service delivery, blended financing mechanisms may enlarge the market and potentially contribute to increased access while enhancing accountability and viable sanitation businesses (11, 69, 79).

In addition to limited awareness of regulations governing sanitation facilities construction, the findings of this study, especially the rarity of outcomes and the variation of random cluster effects, suggest that there is a presence of other underlying behavioral factors across different levels of the sanitation sector, which may be associated with the low full compliance with the selected objectives of the standard. Awareness was proven to be one of the most important factors influencing sanitation behavior change positively; however, its effectiveness depends on deeper community engagement and a nuanced understanding of local context and behavioral triggers that shape sanitation habits (70, 79). The tendency to use one-size-fits-all pit latrines and cesspit facilities observed in this study may highlight the need for diversified designs that reflect the needs of the investigated demographic, in line with Citywide Inclusive Sanitation Principles (71).

Findings of this study further showed that full compliance of pit latrines with selected objectives of RS ISO 24521 was low, which also seems to be a challenge shared with other cities in sub-Saharan Africa. A review study of pit latrines in Sub-Saharan Africa found that pit latrines were not performing well due to bad odors and a lack of cleanliness (42, 45, 72, 73). Another study conducted in Tanzania showed that poorly designed and operated containment (unlined pits, solid trash in the pit) negatively affected containment strength and safe desludging (42). Whereas in this study, no direct association was found between low sustainability of pits and demographic characteristics of surveyed households, another study in Tanzania found that contextual factors (inaccessible pits, inefficient regulations, lack of monitoring) and technological factors (affordability, house tenure, available technologies) contributed to unhygienic pit desludging practices (74). This also accords with Strande (67), who suggests that such containments are not fit for urban areas because they require adequate land availability for treatment and controlled leaching to the soil. Issues related to difficult access to households and sanitation facilities for emptying services, shared sanitation facilities, accountability of landlords, unsustainability of pit latrines and cesspits, unhygienic facilities, and limited inspections may reflect a need to address sanitation issues in a multi-sectoral and faceted approach, including environmental health, urban planning, sanitation infrastructure, and policy implementation (82).

These findings corroborate the National Sanitation Policy (31), which provides high-level guidelines addressing most issues raised by this study except for sanitary inspection. This further emphasizes the need for policy enforcement and the regularization of sanitary inspection as prescribed in the sanitation safety planning developed by WHO (25).

### 4.4 Recommendations

While achievements were made in access to basic sanitation, this study suggests that many households in Kigali may be using pit latrines and cesspits that are poorly compliant with the four selected objectives of RS ISO 24521. Therefore, to improve the compliance of household sanitation facilities with RS ISO 24521 in Kigali, the following recommendations are proposed:

1. *Development of contextualized sanitation solutions* that comply with standards, including inclusive sanitation facilities and financing schemes that provide various choices and address issues such as depths, lining, sharing, odors, and access for emptying, while being driven by quality, acceptance, and affordability. These solutions should be contextualized to meet income levels, residence and settlement type, and water supply dynamics within Kigali city. This will contribute to increasing demand for emptying services.
2. *Enforcement of regulations:* while regulations are available, they need to be adjusted to contextualized facilities and community strata (income level, water access, residence, and settlement type) to instill sustainable adoption. For example, adjusted regulations for new buildings and existing buildings can help regulatory implementation and progressively improve sanitation access. Adjusted regulations should target, among other characteristics, unlined pits, defective superstructure, slabs, pit latrines shared by many households, and tenancy agreements in regard to access to sanitation services.
3. *Sanitary inspections by competent authorities:* continuously inspecting sanitation facilities against standards and delivering corrective measures could help in enforcing regulations and promoting hygiene behaviors. For example, inspections should be done systematically regardless of income, residence, and settlement type of households
4. *Sustainable water supply:* increasing access to piped water and minimizing water shortages in all communities, to enable adequate sanitation and hygiene practices.
5. *Promote sanitation and hygiene behavior changes:* to ensure the effectiveness of facilities and regulations, it is essential to actively involve the community from different strata in defining and selecting measures (for instance, sanitation technologies and related regulations) that promote their health and wellbeing. Moreover, sanitation behavior change can be integrated within policy implementation and broader systems of sanitation services delivery by focusing on increased awareness, triggers, and nudges. Sanitation behavior change can, in turn, facilitate sanitation inspections, inclusive technical solutions and regulations, and adoption of improved sanitation facilities.

### 4.5 Refined assessment tool to evaluate households’ onsite sanitation facilities

The tested assessment tool was used to evaluate the compliance of pit latrines and cesspits with four selected objectives of RS ISO 24521; however, it does not imply that a full compliance assessment was conducted. To be used for such an evaluation, the tool would need to be extended to cover all objectives of the standard. Additionally, some characteristics, such as the availability of a handwashing station and leaks into the environment, were assessed only for pit latrines, even though they also can apply to flush toilets. To improve the effectiveness of the collected information, characteristics such as the presence of a ventilation system, segregation of responsibilities about toilet maintenance for houses under rent, and potential leakage to nearby natural water resources should be considered.

Moreover, some of the reviewed characteristics are likely to have reliability issues. Self-reported data, such as income level, cleanliness, satisfaction, and safety of the facility, may be biased by the enumerator or respondents, and, as sanitation is frequently related to stigma or pride, cultural taboo on sanitation may have led to both under- and over-reporting. Other characteristics that can be difficult to report accurately were not measured or verified, such as groundwater levels and frequency of emptying. Regarding the knowledge of regulations, in addition to being self-reported, only the participant’s response was considered; however, other members of the household may be aware of the regulations.

Considering these limitations, Table 12 presents an improved version of the tool for the assessment of compliance of pit latrines and cesspits for the four objectives of the standard. In this version, some characteristics were removed to simplify routine inspections and focus on easily measurable characteristics, namely, the Presence of gas stench, Reasons for choosing the type of sanitation facility, and Sanitary inspections by authorities. The tool also provides optional information that can be gathered by authorities to complement the understanding of sanitation practices.

**Table 12:** Refined tool with proposed list of key characteristics to investigate.

Topic	Characteristic *Question **Observation	Measurement scale	Standard compliance criteria
Standard objectives			
Protection of Public Health	Evidence of leaking into the surrounding environment**	Yes, No	No occurrence
	Cleanness of the interface and the pit surface**	Clean, Fairly clean to very dirty	Clean
	Presence of flies**	No flies, Few flies, Many flies	No flies
	Pit smell**	No bad smell, Slight to strong bad smell	No bad smell
	Presence of handwashing **	Yes, No	Yes
	Access to water*	Scarce, Intermittent supply, Always available	Always available
Meeting Needs and Expectations of Users	The facility provides security to intended users**	Yes, No	Yes
	Facility sharing status*	Shared, Not shared	Not shared
	Users’ satisfaction with the facility*	Yes, No	Yes
	Danger of the facility to other houses (leaking, risks of collapse, etc)**	Yes, No	No
Sustainability	Type of Slab**	Reinforced concrete, Other waterproof strong material, Other unimproved materials	Reinforced concrete, Other waterproof strong material
	Pit lining status*	Lined, Partially Lined, Unlined	Lined
	Conditions of superstructure **	Good condition, Poor to Very poor condition	Good Condition
	Conditions of the slab and pit**	Good condition, Poor to Very poor condition	Good Condition
	Frequency of emptying*	New, Once or many times, Never	New and Once or many times
	Rainwater drainage into the pit**	Yes, No	No
	Type of effluents and waste discharge into the pit**	Only excreta/Blackwater, Domestic Trash, Laundry, and Kitchen Water	Only excreta/Blackwater
	Presence of an opening for emptying services**	Yes, No	Yes
	Accessibility of the residence by a road at least 3 m wide**	Yes, No	Yes
	Accessibility of the pit for emptying services**	Yes, No	Yes
	Knowledge of regulations about toilet construction in Kigali City*	Yes, No	Yes
Protection of Environment	Depth of the pit*	Less than 6 m, Between 6 and 10 m, Over 10 m	Less than 6 m
	Access to groundwater or moist soil when digging the pit*	Groundwater reached, Moist soil reached, No water reached	No water reached
Demographic and other information			
Demographic and other information	Level of income	Very Low, Low, Middle, High	–
	Residential type*	Compound with many households, Single house	–
	Type of settlement**	Old, New	–
	House tenure*	Owning, Renting	–
	Number of households sharing one pit*	Number	–
	Age of pit*	Less than 5 years, 5–10 years ago, More than 10 years	–
	Recommendations for improved sanitation services*	Open question	–

*Question, **Observation.

In addition to supporting compliance assessments, applying this improved tool can help in strengthening integrated data-driven decision-making across the entire sector, for instance, by using the collected information to identify gaps in sanitation facilities and practices and develop solutions accordingly. However, additional statistical analysis, for instance, the use of GLMM and Rao-Scott adjusted Chi-square tests, were used in this study to deepen the understanding of factors associated with full compliance, and might not be necessary for routine use of this tool.

Last, this study recommends a multisectoral consultation approach to review and validate the proposed assessment tool for pit latrines and cesspits compliance with RS ISO 24521, and to extend the tool-development approach to other types of onsite sanitation facilities that were not covered by this study, such as septic tanks, public and institutional sanitation facilities, and decentralized wastewater treatment plants. A multisectoral approach would facilitate inclusion of inputs from stakeholders and promote ownership and implementation by integrating both structural sustainability, public health, and environmental protection (75). However, the outcomes are likely to be context dependent (43, 75, 80).

### 4.6 Limitations of the study

The all-or-nothing compliance threshold requiring all criteria within a standard objective to be met simultaneously in this study is stringent, and compliance was low for several standard objectives. The statistical analysis (GLMM) did not include a structured analysis of other compliance levels. It focused on cases of Full compliance, thereby leaving scope for subsequent investigations into intermediate compliance levels that were not encompassed within the GLMM analysis. The rarity of outcomes may have affected the stability of the GLMM, requiring cautious interpretation. For that reason, the model was not significant for the standard objectives, Protection of Public Health (for pit latrines) and Sustainability (for both pit latrines and cesspits). Moreover, the comparison between the compliance of pit latrines and cesspits is partial due to the difference in the number of characteristics used for the objectives Protection of Public Health and Sustainability.

The study was limited to Gasabo District and excluded households using septic tanks or sewered systems (~15%), introducing selection bias and limiting generalization to the full Kigali sanitation profile. Additionally, several variables were self-reported and susceptible to recall bias; direct environmental measurements were not conducted, and only four of the seven RS ISO 24521 objectives were assessed, meaning that findings reflect compliance with selected objectives only, not full standard compliance. The tool went through rigorous internal quality refinement; however, no formal content validity index, inter-rater reliability assessment, or agreement statistics were performed. Therefore, a formal multisectoral validation process may need to be undertaken before full adoption of the tool.

## 5 Conclusion

The standard compliance assessment tool developed and tested in this study showed that households with pit latrines had lower compliance with selected objectives of RS ISO 24521 than cesspits. Although some characteristics differed by facility type, pit latrines complied lower with the standard objectives Protection of Public Health and Meeting Needs and Expectations of Users, while both types of facilities had low compliance with Sustainability and Protection of Environment objectives. Low compliance may be linked to the technical characteristics of pits, inadequate sanitary inspections by authorities, and a lack of awareness of regulations. Additionally, limited sanitation solutions in terms of technologies and financing schemes, as well as unsustainable water supply, may be linked to one-size-fits-all solutions and pits with very low sustainability. For example, findings show that, despite being widely used, the majority of users are not satisfied with pit latrines. Moreover, income was found to be an important factor that may be linked to compliance with meeting users’ expectations, protection of public health and environment, with low-income users using facilities that are likely not to comply. Households in shared compounds, or using shared pit latrines, were likely to have low income.

To improve household onsite sanitation services, it is recommended to develop context-specific sanitation technologies addressing each of the factors that were associated with not complying with the selected objective of the RS ISO 24521. It is also recommended to develop financing mechanisms that promote inclusive access and adherence to established standards, alongside strengthened awareness and enforcement of regulations governing household sanitation facilities and landlord agreements. Continuous and systematic sanitary inspections should be implemented, complemented by intensified promotion of sanitation and hygiene behavior change, including handwashing, investment in sustainable toilets, and proper maintenance practices. Findings further highlight the need to enhance behaviorally informed and data-driven decision-making across the sanitation sector and to improve the reliability of water supply services. For example, income level, house tenure, residential types, settlement type, and access to piped water were associated with different compliance characteristics, suggesting that they are important factors to consider when developing sanitation solutions and regulations.

This study also shows that the refined assessment tool is useful for evaluating the compliance of pit latrines and cesspits with selected objectives of RS ISO 24521. Key limitations in the tool include partial coverage of sanitation systems and reliance on self-reported or difficult-to-verify data. An improved version of the tool was therefore proposed to support routine inspections and enhance data-driven decision-making. The study further recommends a multisectoral consultation process to validate and extend the tool to other types of sanitation systems, such as septic tanks and decentralized wastewater treatment systems. The tool is scalable to other cities that have rapid urban growth and extensive reliance on pit latrines and cesspits. Required adaptations include recalibrating compliance criteria to local standards, contextualization of demographic information, and translating the questionnaire into local languages.

## Data Availability

The raw data supporting the conclusions of this article will be made available by the authors, without undue reservation.
